# Macrophages derived from pluripotent stem cells: prospective applications and research gaps

**DOI:** 10.1186/s13578-022-00824-4

**Published:** 2022-06-20

**Authors:** Irina Lyadova, Andrei Vasiliev

**Affiliations:** Koltzov Institute of Developmental Biology of RAS, Moscow, Russian Federation

**Keywords:** Induced pluripotent stem cells, Macrophages, Macrophages derived from induced pluripotent stem cells, Disease modeling, Cell therapy, Host–pathogen interactions

## Abstract

Induced pluripotent stem cells (iPSCs) represent a valuable cell source able to give rise to different cell types of the body. Among the various pathways of iPSC differentiation, the differentiation into macrophages is a recently developed and rapidly growing technique. Macrophages play a key role in the control of host homeostasis. Their dysfunction underlies many diseases, including hereditary, infectious, oncological, metabolic and other disorders. Targeting macrophage activity and developing macrophage-based cell therapy represent promising tools for the treatment of many pathological conditions. Macrophages generated from human iPSCs (iMphs) provide great opportunities in these areas. The generation of iMphs is based on a step-wise differentiation of iPSCs into mesoderm, hematopoietic progenitors, myeloid monocyte-like cells and macrophages. The technique allows to obtain standardizable populations of human macrophages from any individual, scale up macrophage production and introduce genetic modifications, which gives significant advantages over the standard source of human macrophages, monocyte-derived macrophages. The spectrum of iMph applications is rapidly growing. iMphs have been successfully used to model hereditary diseases and macrophage-pathogen interactions, as well as to test drugs. iMph use for cell therapy is another promising and rapidly developing area of research. The principles and the details of iMph generation have recently been reviewed. This review systemizes current and prospective iMph applications and discusses the problem of iMph safety and other issues that need to be explored before iMphs become clinically applicable.

## Introduction

Since the pioneering research by S. Yamanaka’s laboratory [1] that described transcriptional factors able to convert mature somatic cells into pluripotent cells, iPSCs started to be widely used to generate a wide variety of specialized cell types. One of the actively developed directions is the differentiation of iPSCs into innate immune cells, particularly, into macrophages.

Macrophages play a key role in the control of host homeostasis. The function relies on the high phagocytic and secretory activities of macrophages, which underlie their capacity to eliminate invading pathogens, clear dead and transformed self-cells, induce inflammatory reactions, as well as control exaggerated immune responses, and mediate tissue repair [2–5]. The multifaceted actions of macrophages depend on their capacity to sense the surrounding milieu and fine-tune their own functional state in such a way as to regulate the changing environment. Depending on the environmental cues, macrophages may acquire various multiple states, which simplistically are usually divided into two main types, M1-like or pro-inflammatory and M2-like or anti-inflammatory (or alternatively-activated). M1-like macrophages are formed in response to IFN-γ, TNF, granulocyte–macrophage colony-stimulating factor (GM-CSF) and various pathogen-derived signals (primarily, lipopolysaccharide (LPS)); the cells produce elevated levels of pro-inflammatory factors, such as TNF, IL-1β and IL-12, implement effective antigen presentation and costimulation, as well as mediate protective anti-infectious and anti-cancer responses. M2-like macrophages develop under the action of macrophage colony-stimulating factor (M-CSF), IL-4, IL-13, IL-10, TGF-β, immune complexes, glucocorticoids or a combination of some of these stimuli; depending on the inducing factors, different subtypes of M2-like macrophages are identified. The cells produce factors favoring Th2 response, mediate wound healing and tissue regeneration (for a detailed review of the current understanding of M1-M2 paradigm see [6–12]). Serious impairment of macrophage functions or disbalance between their pro- and anti-inflammatory activities underlie the pathogenesis of many diseases, including infections, cancer, cardiovascular, metabolic, neurodegenerative, autoimmune and hereditary disorders [9, 13–19]. This makes macrophages an attractive therapeutic target and a promising cell therapy tool and sets the task of developing adequate approaches to generating macrophages and learning how to expand their production ex vivo and to modulate their activity in a desired way.

Until recently, macrophages derived from peripheral blood monocytes (monocyte-derived macrophages, MDMs) were the main source of human macrophages [20]. Lately, approaches to generating macrophages (iMphs) from pluripotent stem cells, either induced or embryonic, were developed and were shown to have several advantages over the MDM model (discussed below). Over the last few years, the number of studies aimed at iMph generation and analysis has been rapidly growing, leading to the appearance of new effective protocols of iMph differentiation, a deeper understanding of iMph biology and the emergence of new promising iMph applications. Recently, we have reviewed the principles and methodological aspects of iMph generation [21]. Here, we systemize the most actively developed areas of human iMph studies and discuss the prospects and limitations in this context.

## Principles of iMph generation and the advantages of the model

The principles of iMph generation rely on the iPSC capacity to give rise to different germ layers, including mesoderm. Once mesoderm is formed, the cells are cultured in conditions that favor the generation of hematopoietic progenitors (HPPs), their myeloid specification and iMph terminal differentiation. To drive the iMph differentiation process, various protocols have been elaborated and are currently used (reviewed in [21]). Their variety can be reduced to four main types.

In *OP9-based protocols*, iPSCs are cultured in the presence of mouse bone marrow OP9 stromal cells until HPPs are generated; HPPs are then cultured in the presence of M-CSF or GM-CSF to induce myeloid specification and iMph formation [22–25]. The use of xenogeneic stromal cells reduces the reproducibility and clinical applicability of the protocols, and therefore OP9-independent protocols are currently preferred.

**Fig. 1:**
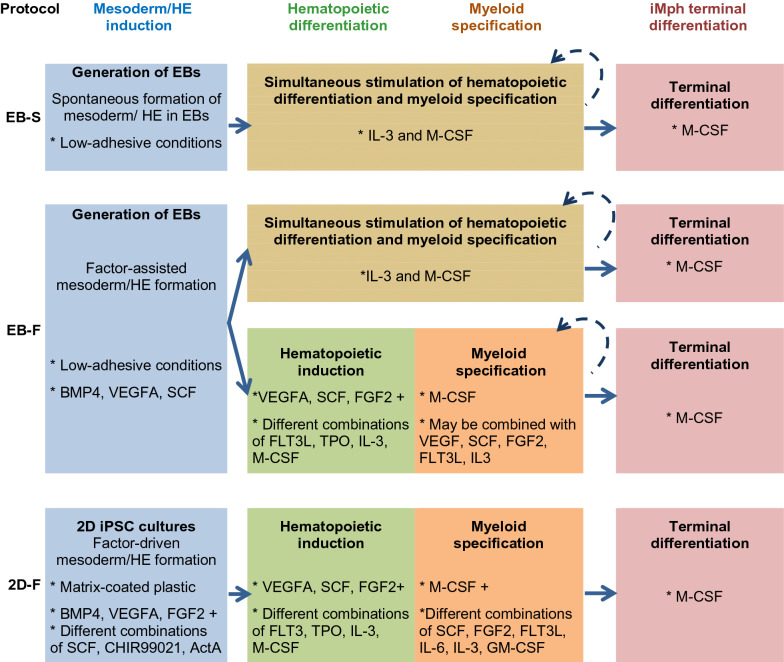
Principles of iMph differentiation used in different OP9-independent protocols. iPSC differentiation into iMphs goes on through four main stages: the induction of mesoderm and hemogenic endothelium (HE), the induction of hematopoietic differentiation, myeloid specification of hematopoietic progenitors and terminal differentiation of the generated monocyte-like cells into iMphs. In EB-S protocols, to induce mesoderm and HE, iPSCs are cultured in low-adhesive conditions, which stimulate the formation of 3D cell aggregates, embryoid bodies (EBs). Within the EBs, mesoderm and HE are generated spontaneously, due to the tight intercellular interactions. After EBs are formed, they are transferred to tissue culture (TC) plates and cultured in the presence of IL-3 and M-CSF that induce the formation of hematopoietic progenitors and their myeloid specification. When monocyte-like cells appear in the culture, they are transferred to new TC plates, where their terminal differentiation into iMphs is directed by M-CSF. The remaining cultures are restimulated with IL-3 and M-CSF to induce new rounds of myeloid cell generation. In EB-F protocols, mesoderm/HE are also induced through the formation of EBs. However, differently from EB-S protocols, exogenous factors are added to the cultures to support the mesodermal pathway of cell differentiation. This increases the reproducibility and the efficacy of the protocols. Subsequent stages are induced either by culturing EBs in the presence of IL-3 and M-CSF (like in EB-S protocols) or by adding mixes of exogenous factors, that sequentially drive the cells through the hematopoietic and myeloid differentiation stages. 2D-F protocols do not imply EB formation. iPSCs are cultured in TC plates, where complex mixes of exogenous factors are sequentially added to drive the cells through the differentiation process. Color clues: Blue, mesoderm/HE induction; Green, hematopoietic differentiation; Orange, myeloid specification; Green/orange shaded, hematopoietic and myeloid differentiations are induced simultaneously; Pink, iMph terminal differentiation. Asterisk, exogenous factors and other special conditions used at the indicated stages. *BMP4* Bone Morphogenetic Protein 4, *CHIR99021* GSK inhibitor/Wnt activator, *DKK-1* Wnt inhibitor, *EB* embryoid body, *FGF2* basic fibroblast growth factor, *FLT3L* FMS-like tyrosine kinase 3 ligand, *HE* hemogenic endothelium, *IL-3* interleukin-3, *IL-6* interleukin-6, *SCF* stem cell factor, *TPO* thrombopoietin, *VEGFA* Vascular Endothelial Growth Factor A

In OP9-independent protocols, mesoderm and hemogenic endothelium (HE) are induced using two different approaches, i.e., through the formation of embryoid bodies (EBs) or EB-independently (Fig. 1).

EBs are 3D cell structures capable of differentiating into all three germ layers, i.e., ectoderm, mesoderm and endoderm. To generate EBs, iPSCs are cultured in low-adherent conditions which favor cell–cell interactions and EB formation. Within EBs, mesoderm and HE may form spontaneously, in the absence of any exogenously added factors (*“spontaneous” EB-based protocols, EB-S*) [26–33]*. *However, to enhance mesoderm/HE formation and increase the efficacy of iMph generation, some authors perform EB formation in the presence of exogenous mesoderm/HE inducing factors, such as BMP4, VEGFA and SCF (*EB-based factor-assisted protocols, EB-F*). The subsequent generation of HPPs and myeloid monocyte-like cells may be driven by culturing EBs in the presence of only two cytokines, IL-3 and M-CSF [27, 34–36], or by adding more complex mixes of exogenous factors, which sequentially lead the cells through the hematopoietic and myeloid differentiation stages (e.g., VEGFA, SCF, FGF2, FLT3L, TPO, IL-3, M-CSF) and result to the formation of monocyte-like cells [37–39].

Some protocols induce mesoderm without forming EBs (*2D factor-dependent protocols, 2D-F)*. In these protocols, iPSCs are cultured on matrix-coated plates, and all differentiation stages, starting from the stage of mesoderm formation, are induced by multiple exogenous factors (such as BMP4, CHIR99021, Activin A, VEGFA, FGF2, SCF, IL-6, IL-3, M-CSF et al.). These factors, being added to the cultures sequentially and in different combinations, drive cells through mesoderm/HE --> HPPs --> myeloid cell differentiation pathway [40–43].

In most types of protocols, the last differentiation stage consists in the cultivation of myeloid cells in the presence of M-CSF and results in the formation of terminally differentiated iMphs [26, 27, 29–43].

iMphs obtained using different protocols have a typical macrophage morphology (large vacuolated cells with pseudopodia), express a typical macrophage phenotype (CD14^+^CD11b^+^CD45^+^) and execute the main macrophage functions, such as phagocytosis and the secretion of pro- and anti-inflammatory cytokines (reviewed in [21, 44] and [27–29, 37, 45–47]).

Because iPSCs can easily be obtained from adult somatic cells and have a high self-renewal capacity, the differentiation of macrophages from iPSCs potentially allows to obtain iMphs from any individual, of any genetic background, in unlimited quantities and it also allows to standardize iMph populations. Furthermore, it is relatively easy to edit iPSCs genetically, which makes it possible to generate genetically manipulated iMphs. Other advantages of iMphs include their scalability and potential clinical applicability. In this context, there are differences between different types of protocols, which need to be briefly summarized (reviewed in detail in [21]).

EB-S protocols are relatively cheap and least labor-intensive, but they have insufficient reproducibility due to a poor control of the initial differentiation stage. Additionally, they are not clinically applicable due to the use of xeno-dependent and chemically-undefined conditions. 2D-F protocols provide full control over the differentiation process and are clinically applicable due to the use of xeno-free and chemically defined conditions. However, they are labor-intensive, highly expensive and difficult to scale up as they are based on adhesive cultures. EB-F protocols, especially those in which myeloid differentiation is driven by only two factors, IL-3 and M-CSF, combine the main advantages of EB-S and 2D-F approaches, i.e., reproducibility, clinical applicability, cost and labor efficacy. The yield of the resulting iMph population is another important parameter to consider. Unfortunately, so far, yield efficacy of different protocols has not been compared side-by-side. However, EB-S and EB-F protocols have been shown to be scalable to bioreactor [46] and spinner [35] cultures, which is an important benefit, especially for clinical applications.

Overall, the availability, scalability, standardizability and editability of iMphs make them suitable for a wide variety of applications. Those applications that are being developed most actively are examined below. For each study discussed in the present review, the protocols used for obtaining iMphs are carefully indicated in brackets.

## Hereditary disease modeling

Monocyte/macrophage dysfunction underlies a number of rare inherited disorders. However, samples from patients with rare diseases are difficult to obtain. This hampers both disease pathogenesis analysis and the search for new therapeutic targets. The elaboration of strategies for generating iMphs has provided a unique opportunity to generate mutation-affected macrophages, accumulate them in unlimited numbers and create cell models of phagocyte-associated hereditary diseases (Table 1). This is achieved either by generating patient-derived iPSCs or by introducing mutations into iPSCs derived from healthy donors followed by iPSC to iMph differentiation.

**Table 1:** The use of iMphs for hereditary disease modeling and drug testing

Target disease	Reference	iPSC/iMph source (mutation)	iPSC/iMph genetic modification performed in the study	iMph characteristics and other results
GD	Panicker et al. [26]	Patients with type 1, 2 and 3 GD	–	GD-iMphs: a low GBA1 enzymatic activity; an accumulation of sphingolipids in the lysosomes; a defective RBC clearance iMph capacity to clear RBCs was fully restored by recombinant GBA1 and partially restored by isofagomine
GD and PD	Aflaki et al. [56]	Type 1and type 2 GD patients with and without parkinsonism	–	GD-iMphs: a decreased GBA1 activity; glucosylceramide and glucosylsphingosine are stored in iMphs GD-neurons: a reduced dopamine transporter reuptake; an elevated α-synuclein levels NCGC607 drug restored GBA1 activity iMphs and reduced SNCA levels in dopaminergic neurons generated from iPSCs derived from GD patients with Parkinsonism
PD	Haenseler et al. [54]	Patients with early-onset PD (A53T or SNCA triplication)	–	PD-iMphs: an increased intracellular SNCA; a higher release of SNCA; a reduced phagocytic activity
PD, NCL, RS	Munn et al. [57]	Healthy donor	Introduced mutations: SNCA A53T; GRN2/GRN R493X; MECP2-КO	Engineered iMphs: a typical macrophage phenotype; an impaired phagocytic function; some transcriptomic and secretory differences compared to parental iMphs. Detailed comparison of live and cryopreserved iMphs was performed
CGD	Jiang et al. [60] Brault et al. [25]	Patients with CGD (gp91^phox^, AR p47^phox^ or p22^phox^ deficiencies)	–	CGD-iMphs: an impaired production of ROS; the cells can be cryopreserved
	Klatt et al. [62]	Healthy donor; CGD patient (p47^phox^-deficiency)	Introduced mutations: p47-ΔGT –	p47-ΔGT-iMphs and CGD-iMphs: an impaired bacteria killing (*E. coli);* the function was restored after the correction of the mutation
	Flynn et al. [61]	CGD patient (gp91^phox^ intronic mutation)	CRISPR/Cas9 gene correction	CGD-iMphs: a hampered oxidative burst, restored following gene correction
FMF	Takata et al. [41]	FMF patient (homozygous p.Met694Val mutation of *MEFV*)	–	FMF-iMphs: an increased secretion of IL-1β, IL-18, TNF-α, CCL4 in response to LPS
TD	Zhang et al. [37]	TD patients (heterozygote at S2046R/K531N; homozygous E1005X/E1005X truncation)	–	TD-iMphs: a defective cholesterol efflux; an increased response to LPS compared to control iMphs (*IL1B, IL8, TNF, CCL5)*
	Gupta et al. [66]	Healthy donor	Frameshift in ABCA1 gene (CRISPR/Cas9)	Engineered iMphs: a reduced cholesterol efflux; a higher IL-1β production; a higher response to LPS (*IL1B*, *IL8*, and *CCL5)* compared to isogenic control iMphs
BS	Takada et al. [68]	Healthy donors BS patients	Introduced mutation: NOD2 R334W –	BS-iMphs and engineered iMphs: an enhanced inflammatory response to IFN-γ
PAP	Suzuki et al. [70]	Children with hereditary PAP		PAP-iMphs: an impaired GM-CSF receptor signaling; a reduced expression of GM-CSF receptor dependent genes; an impaired surfactant clearance
IBDs	Mukhopadhyay et al. [71]	IBD patient (homozygous splice site mutation of *IL10RB*)	–	IBD-iMphs: cell overactivation; a hampered antibacterial control (*S. typhimurium*); overexpression of genes involved in PGE2 biosynthesis; an increased PGE2 production
	Sens et al. [73]	Healthy donor Very-early onset IBD patient	KO:*IL10RA, IL10RB, STAT1, STAT3* *-*	Engineered iMphs and IBD-iMphs: IL-10 fails to suppress LPS-induced inflammatory response
CINCA	Tanaka et al. [75]	Patients with mosaic CINCA	-	CINCA-iMphs: abnormal production of IL-1β; cells are susceptible to LPS-induced pyroptosis; inhibitors of NLRP3 pathways reduced IL-1β secretion
AD NHD	McQuade et al. [80]	Healthy donors	TREM2 knockout	Engineered iMGs: a decreased cell survival; a reduced phagocytosis of apolipoprotein E and β-Amyloid; a reduced chemotaxis to SDF-1α; an impaired in vivo response to β-Amyloid
	Reich et al. [84]	Control iPSCs* TREM2-KO iPSCs*	-	TREM2-KO iMGs: a stronger migration towards C5e complement; a stronger increase in intracellular Ca in response to danger signals
	Hall-Robets et al. [85]	Control iPSCs* R47H iPSCs* TREM-KO iPSCs*	-	TREM2-KO iMGs: impaired survival, motility, phagocytosis R47H iMGs: a reduced adhesion to vitronectin; disregulation of genes involved in cell proliferation, adhesion, motility, immunity
	Piers et al. [82]	Control iPSCs* R47H^het^ iPSCs* R47H^hom^ iPSCs*	-	R47H iMGs: a respiratory deficit; an impaired switch to glycolysis following immune challenge; a hampered phagocytosis of β-Amyloid. PPARγ agonist normalizes glycolysis switch and phagocytosis
	Cosker et al. [83]	Control iPSCs* R47H^het^ iPSCs* R47H^hom^ iPSCs*	-	R47H iMGs: a reduced SYK signalling and a reduced NLRP3 inflammasome response upon cell stimulation with TREM2 ligand phosphatidylserine
	Garcia-Reitboeck et al. [81]	NHD patients (T66M/T66M, W50C/W50c)	-	NHD-iMGs: reduced expression/secretion of TREM2 and iMG survival; an impaired phagocytosis of apoptotic bodies

*AD* Alzheimer’s disease, *AR* autosomal recessive, *BS* Early-onset sarcoidosis or Blau syndrome, *CGD* chronic granulomatous disease, *CINCA* chronic infantile neurologic cutaneous and articular syndrome, *FMF* Familial Mediterranean fever, *GD* Gaucher disease, *GRN* Progranulin, *IBD* inflammatory bowel diseases, *iMGs* iPSC-derived microglia, *MeCP2* methyl-CpG-binding protein 2, *NCL* Neuronal ceroid lipofuscinosis, *NHD* Nasu-Hakola disease, *NLRP3* NOD-, LRR- and pyrin domain-containing protein 3, *PD* Parkinson’s disease, *PGE2* prostaglandin E2, *RBCs* red blood cells, *RS* Rett syndrome, *SNCA* Αlpha-synuclein, *TD* Tangier disease, *TREM2* Triggering receptor expressed on myeloid cells 2

### Gaucher disease (GD)

GD is an autosomal recessive (AR) disorder caused by mutations in the β-glucocerebrosidase gene (*GBA1*). Normally, GBA1 cleaves glucose moiety from glucosylceramide and glucosylsphingosine in the lysosomes. Mutations lead to the accumulation of glucosylceramide and glucosylsphingosine lipid substrates within the lysosomes of macrophages, the accumulation of engorged macrophages in different organs, the activation of pro-inflammatory macrophage responses and the development of GD. The latter manifests with variable visceral, hematological and skeletal symptoms including splenomegaly, thrombocytopenia, neuropathy, osteonecrosis, osteoporosis, fractures, predispositions to malignancy, Parkinson’s disease and other pathologies [48]. Depending on the clinical manifestations and neurological involvement, 3 types of GD are identified, of which type 1 affects viscera, while type 2 and type 3 are characterized by neuropathy, severe in type 2 and variable in type 3 [49]. Available disease-specific treatments include enzyme replacement and substrate reduction therapies [50]. However, they are not effective to treat type 2 and type 3 GD neuropathy. Future prospects are associated with cell and gene therapy, the development of which requires adequate disease models [51].

In 2012 Panicker and co-authors [26] differentiated iMphs from iPSCs derived from type 1, type 2 and type 3 GD patients using an EB-S protocol. GD-iMphs expressed key macrophage characteristics and recapitulated the main phenotypic hallmarks of GD. Specifically, they exhibited low GBA1 enzymatic activity; accumulated sphingolipids; expressed elevated levels of TNF-α, IL-6 and IL-1β cytokines and displayed a defective clearance of phagocytosed red blood cells (RBCs) [51]. Of note, the kinetics of RBC clearance by iMphs correlated with the severity of the mutations. iMph treatment with recombinant GBA1 restored RBC clearance, supporting the suggestion that the defects of GD-iMphs were indeed caused by GBA1 deficiency [26]. In another study, GD-iMphs (2D-F) modeled necroptosis, a pathway implicated in the development of GD-associated neuroinflammation. Specifically, iMphs generated from healthy donors and treated with GBA1 inhibitor or derived from GD patient displayed an altered growth potential and an increased expression of necroprosis-associated genes *RIPK3* and *MLKL* [52].

### Parkinson’s disease (PD)

PD is a neurodegenerative disorder characterized by an intracellular accumulation of α-synuclein aggregates and the formation of Lewy bodies in the brain. Mutations in the α-synuclein (*SNCA)* gene promote α-synuclein accumulation and the development of early-onset PD [53]. To better understand the mechanisms of α-synuclein aggregation, Haenseler and co-authors [54] developed an iMph-based model in which iMphs (EB-F) were generated from early-onset PD patients bearing *SNCA* A53T or *SNCA* triplication mutations. iMphs with *SNCA* triplication had significantly increased intracellular α-synuclein, released significantly more α-synuclein into the medium and exhibited a significantly reduced phagocytosis, thus recapitulating the donor phenotype.

It has been reported that α-synuclein and GBA1 are in inverse relationships, and that GD patients have an increased risk of developing PD [55]. However, the mechanisms underlying these associations are incompletely understood. To get an insight into these mechanisms, Aflaki and co-authors [56] generated iPSCs from type 2 and type 1 GD patients with and without Parkinsonism and differentiated them into iMphs (EB-S) and neurons. GD-iMphs had a decreased GBA1 activity and stored glucosylceramide and glucosylsphingosine; GD-neurons derived from patients with Parkinsonism had a reduced dopamine storage, a reduced dopamine transporter reuptake and elevated α-synuclein levels. As will be discussed below, the model was used to test potential therapeutic drugs.

Munn and co-authors [57] modeled several neurodegenerative disorders, including PD, by introducing mutations SNCA A53T (PD-associated), GRN2/GRN R493X (associated with neuronal ceroid lipofuscinosis) and MECP2-Knockout (responsible for Rett syndrome) to the same isogenic iPSC line and differentiating the modified iPSCs to iMphs (EB-F). All iMphs expressed macrophage-specific markers, were phagocytic and responded to specific stimuli. It was concluded that the introduction of disease-associated mutations into iPSCs followed by iPSC differentiation into iMphs creates a relevant model to study molecular pathways of inflammation associated with neurodegeneration.

### Chronic granulomatous disease (CGD)

CGD is a rare inherited immunodeficiency characterized by the inability of phagocytes to generate microbicidal reactive oxygen species (ROS) and to kill engulfed pathogens. The disease develops as a result of mutations in any of the 5 genes encoding the nicotinamide-adenine-dinucleotide-phosphate (NADPH) oxidase complex, i.e., *CYBB* (encodes the gp91^phox^ protein and causes X-linked CGD), *CEBA, NCF1, NCF2* and *NCF4* (encode p22^phox^, p47^phox^, p67^phox^ and p40^phox^ proteins, respectively, and cause AR CGD) [58, 59]. CGD patients suffer from recurrent life-threatening infections in their lungs, skin, lymph nodes, liver and other areas. Current treatment approaches include antimicrobial/antifungal therapy or bone marrow transplantation, each of which has limitations, thus calling for a better understanding of disease pathogenesis and the development of new effective therapeutic strategies. The tasks require adequate disease models.

The models were created by generating iMphs from patients bearing X-linked gp91^phox^, AR p47^phox^ or AR p22^phox^ deficiencies using OP9-dependent [25] or EB-S [60, 61] protocols. CGD-iMphs had normal phagocytic activity but lacked the production of reactive oxygen species (ROS), which is in line with the known CGD pathogenesis. Gene editing with CRISPR/Cas9 restored oxidative burst function in CGD-iMphs providing a proof-of-principle for CGD gene therapy [61]. In the other approach, a cell model of CGD was created using CRISPR/Cas9 technology: iPSCs were generated from a healthy donor, p47-DGT mutation in the p47^phox^ subunit was introduced using CRISPR/Cas9, and the cells were differentiated into iMphs (EB-S). The latter displayed a decreased capacity to kill phagocytosed GFP-labeled *Escherichia coli* [62].

### Familial Mediterranean fever (FMF)

FMF, a monogenic AR periodic fever syndrome, develops as a result of mutations in the *MEFV* gene. The *MEFV* gene encodes pyrin, an intracellular pattern recognition receptor associated with an inflammasome complex; its mutations lead to an enhanced maturation of IL-1β and an exaggerated inflammatory response characterized by recurrent episodes of fever, arthritis, serositis, and renal complications. When iPSCs derived from an FMF patient carrying homozygous p.Met694Val mutation of the *MEFV* were differentiated to iMphs (2D-F), the latter secreted significantly higher levels of IL-1β, IL-18, TNF-α and CCL4 in response to LPS stimulation compared to iMphs obtained from a heterozygous asymptomatic parent, thus reflecting and modeling pathological processes occurring in vivo [41].

### Tangier disease (TD)

TD, another AR disorder, develops due to mutations in the ATP-binding cassette transporter A1 (*ABCA1*) gene. The pathology is characterized by an impaired cholesterol efflux from macrophages, the absence of high-density lipoprotein cholesterol from plasma and the presence of foam cells throughout the body; clinical manifestations include hepatosplenomegaly, peripheral neuropathy and premature coronary artery disease [63–65]. The disease was modeled by generating iMphs from TD patients (EB-F) [37] or from iPSCs with the introduced frameshift in the *ABCA1* gene (EB-S) [66]. In both cases, iMphs recapitulated the key cellular defects of TD macrophages, including a decreased cholesterol efflux, concomitant metabolic impairments, and an increased production of proinflammatory cytokines.

### Early-onset sarcoidosis or Blau syndrome (BS)

BS is a juvenile-onset monogenic auto-inflammatory systemic granulomatous disease associated with a mutation in the *NOD2* (the pattern recognition receptor, nucleotide-binding oligomerization domain 2) gene. The disease manifests before the age of 4 years and is characterized by granulomatous polyarthritis, dermatitis and uveitis, ultimately leading to severe complications including joint destruction and blindness [67]. Morphological signs include the formation of multinuclear giant cells and granulomas composed of macrophages and lymphocytes. It is understood that the mechanisms underlying BS include the ligation of NOD2 with the cognate ligand (e.g., muramyl dipeptide of bacterial cell wall) and the subsequent activation of the NF-kB pathway. However, the detailed molecular pathways of BS are incompletely understood.

Takada and co-authors [68] generated iMphs (2D-F) from BS patient-derived iPSCs; they also obtained iMphs from iPSCs derived from healthy donor and bearing the introduced BS-associated NOD2 R334W mutation. The models allowed to demonstrate that not only the NOD2 ligand, but also IFN-γ induced an enhanced inflammatory response in BS-iMphs, thus identifying a novel, IFN-γ-dependent, NOD2 ligand-independent mechanism of autoinflammation in BS pathogenesis. One of the outcomes of the findings is the explanation of possible mechanisms underlying the flare-up of BS symptoms after Bacillus Calmette–Guérin (BCG) vaccination, an intervention known to induce IFN-γ.

### Pulmonary alveolar proteinosis (PAP)

PAP is a rare lung disease developed due to an excessive accumulation of surfactant in the alveoli associated with an impaired function of AMs. Three main types of PAP are distinguished, i.e., autoimmune, secondary and hereditary. Autoimmune PAP is characterized by the production of anti-GM-CSF antibodies, which results in GM-CSF deficiency and alveolar macrophage (AM) dysfunction. Secondary PAP results from any disease that affects AMs, most often, it is a result of myelodysplastic syndrome, chronic myelogenous leukemia, acute myeloid leukemia or the inhalation of destructive environmental agents, such as silica, cement etc. In hereditary PAP, AM dysfunction develops due to mutations in alpha- or beta-subunit of the GM-CSF receptor, surfactant protein B or C, ATP-binding cassette 3, or NK2 homeobox 1 (reviewed in [69]). iMphs generated from children with hereditary PAP (OP9-dependent protocol) reproduced defects of AMs seen in PAP. Specifically, PAP-iMphs demonstrated impaired GM-CSF receptor signaling, reduced expression of GM-CSF receptor dependent genes, decreased proliferation in response to GM-CSF stimulation, impaired surfactant clearance and proinflammatory cytokine secretion. Correction of *CSF2RA* gene using lentiviral vector restored surfactant clearance and eliminated other abnormalities, thus confirming the critical role of GM-CSF signaling in surfactant homeostasis and PAP pathogenesis [70].

### Inflammatory bowel disease (IBD)

IBD is a group of complex chronic inflammatory conditions of the gastrointestinal tract. Studies in mice identified deficient IL-10 signaling and macrophage overactivation as critical components of IBD pathogenesis. However, analysis of IBD pathophysiology in humans is difficult due to low sample availability. To address molecular pathways implicated in IBD pathogenesis, several groups generated iMphs from iPSCs derived from IBD patients, very-only onset IBD patients (VEO-IBD) or healthy donors with an introduced knockout of *IL-10RA, IL-10RB, STAT1* or *STAT3* [71]. In all models, IL-10 failed to suppress LPS-induced secretion of proinflammatory cytokines, which was associated with deficient STAT3 phosphorylation and SOCS3 expression [72]. The use of IBD-iMphs also allowed to identify a novel regulatory loop between deficient IL-10 signaling and prostaglandin E2 overproduction and to demonstrate that the mutation leads not only to macrophage overactivation, but also to a hampered macrophage antibacterial control (as shown using *S. typhimurium* intracellular infection [71]).

### Chronic infantile neurologic cutaneous and articular syndrome (CINCA)

CINCA (or neonatal onset multisystem inflammatory disease, NOMID) is a rare inherited autoinflammatory disease developed due to autosomal dominant gain of function mutations in *NLRP3* (NOD-, LRR- and pyrin domain-containing protein 3*).* The mutations lead to systemic inflammation caused by an overproduction of IL-1β and manifested by skin rash, severe arthro- and neuropathy, including contractures, aseptic meningitis, brain atrophy and mental delay [74]. While approximately half of CINCA patients carry heterozygous mutations of the *NLRP3* gene, some patients are carriers of somatic mosaicism. In mosaic patients, the proportion of mutant cells is relatively low, which creates additional difficulties in obtaining mutant cells for the analysis. The mosaicism also raises a question on whether *NLRP3* mutant cells are indeed responsible for the pathology (or the latter is due to other cells bearing yet-unknown mutations). To address the question, Tanaka and co-authors [75] generated mutant and non-mutant iPSCs and iPSC-derived iMphs from two mosaic CINCA patients (OP9-dependent protocol). *NLRP3*-mutant iMphs exhibited abnormal IL-1β production, they were susceptible to LPS-induced pyroptosis and they promoted increased secretion of IL-1β in mosaic cell cultures containing mutant and non-mutant iMphs*.* Inhibitors of the signaling pathways operating upstream and downstream of NLRP3 inflammasome decreased IL-1β secretion by mutant iMphs. The study confirmed the impact of mosaic mutant cells in CINCA pathogenesis and demonstrated the utility of iMph approach for drug screening.

### Alzheimer disease (AD) and microglia

AD, the leading cause of dementia, is a progressive neurodegenerative disorder characterized by β-amyloid deposition and tau hyperphosphorylation [76]. Numerous studies have linked AD (as well as other neurodegenerative conditions associated with the development of dementias) to deficient microglial function (reviewed in [77]). Microglia are brain-resident mononuclear phagocytes that differ from other tissue-resident macrophages by their origin and transcriptomic profile and that play a specific role in the central nervous system development, homeostasis and neuroinflammation [78]. A detailed consideration of microglia biology is beyond the scope of the present review; however, the use of iPSC-derived microglia (iMG) for neurodegenerative disease modeling should be briefly reviewed.

The expression of the Triggering Receptor Expressed On Myeloid Cells 2 (TREM2) is a characteristic feature of microglia. TREM2 is a transmembrane receptor whose ligation induces the phosphorylation of spleen associated tyrosine kinase (SYK) and may affect multiple cell functions including survival, proliferation, metabolism, phagocytosis and chemotaxis [79]. Mutations of TREM2 have been associated with an increased risk of various neurodegenerative disorders, including late onset AD (associated with heterozygous coding variants in TREM2, particularly, R47H), Nasu-Hakola disease and frontotemporal dementia (associated with homozygous missense TREM2 mutations T66M/T66M and W50C/W50C) [80, 81]. The exact role of TREM2 in microglia functionality and neurodegenerative pathologies is not fully clear, which is largely due to a limited access to primary human microglia samples. The generation of iMG cells performed during recent years has allowed to reproduce mutant microglia and to shed some light on these questions.

iMG cells were generated from patients bearing T66M/T66M, W50C/W50C or R47H mutations or obtained from wild type iPSC lines with CRISPR-introduced TREM2 knockout. iMG analyses demonstrated the role of TREM2 in the support of microglia survival [80, 81], mitochondrial respiratory activity and glycolytic immunometabolic switch [82, 83], cell migration and adhesion [80, 84, 85], the development of NLRP3 inflammasome response [83], as well as in cell ability to uptake pathology-associated substrates, such as amyloid-β, apolipoprotein E and apoptotic bodies [80, 81]. Furthermore, it was found that TREM2 deficit dysregulates PPARγ/p38MAPK signaling, and that PPARγ agonist can ameliorate iMG metabolic processes and amyloid-β phagocytosis [82]. Of note, currently, not all iMG data are consistent. For example, TREM2-deficient iMG cells displayed a reduced response to inflammatory stimuli in some [82, 83] but not in other [81, 86] studies; in different studies, different phenotypes appeared as the main result of TREM deficiency (i.e., impaired phagocytosis, metabolism, survival or NLRP3 inflammasome reactivity). Nevertheless, it is anticipated that wide scale use of the iMG model, including iMG in vivo analysis (as it has recently been done by McQuade and co-authors [80]) should help advance our understanding of neurodegeneration mechanisms and microglia biology.

To summarize, iMphs have been successfully used to model hereditary diseases the pathogenesis of which is directly linked to the impairment of main macrophage functions, including substrate degradation in the lysosomes (GD) and the clearance of extracellular substances and dead cells (PAP, TD), macrophage antimicrobial activity (CGD) and inflammation control (FMF, BS. IBD, CINCA). In all models, patient-derived iMphs or healthy donor-derived iMphs with introduced mutations reliably recapitulated the main cellular and molecular features of the diseases, thus creating a unique opportunity to address molecular pathways of disease pathogenesis (including the pathogenesis of rare diseases) using a standardizable cell population. It may be expected that the number of iMph-based disease models will be increasing and will extend to include the modeling of non-hereditary diseases. Of them, those associated with chronic inflammation are particularly attractive. A separate area of research is the use of iMphs for the modeling of phagocyte-pathogen interactions (discussed below).

## Drug testing

Providing an appropriate approach to modeling hereditary diseases, iMphs may also serve as a valuable model to test new drugs and therapeutic approaches. For these purposes, the homogeneity and the scalability of iMphs and their proximity to in vivo persisting macrophages (in contrast to monocyte-macrophage cell lines) are of importance.

Panicker and co-authors [26] used their GD iMph model to compare the efficacy of two GD treatments, recombinant glucocerebrosidase and the chaperone isofagomine. iMph treatment with glucocerebrosidase completely restored iMph capacity to clear RBCs, whereas the treatment with isofagomine restored RBC clearance only partially. The results correlated with the known clinical efficacy of the two drugs confirming the suitability of the iMph model for drug testing.

Previously, a high throughput screening (HTS) assay performed using spleen cell extracts identified several molecules able to restore GBA1 activity [87]. Aflaki and co-authors [56 Aflaki] used GD-iMphs as a more appropriate model (compared to spleen cells) to test the activity of one of the identified drugs, NCGC607. NCGC607 not only restored GBA1 activity, but also reduced α-synuclein levels in dopaminergic neurons generated from iPSCs derived from GD patients with Parkinsonism. Thus, the parallel use of iMphs and iPSC-derived neurons made it possible to identify a potential drug for the two associated pathologies.

IBD-iMphs have impaired IL10 signaling and improper reactivity to LPS. In the aforementioned study by Sens and co-authors [73], IBD-iMph treatment with anti-inflammatory small molecules SB202190 and filgotinib reduced proinflammatory cytokine secretion.

Immortalized monocytic cell lines derived from iPSCs of CINCA patients were successfully used to perform HTS of 4,825 compounds and allowed to identify 7 compounds with IL-1β inhibitory activity. The results have proven the validity of the system for identifying drug candidates to treat monocyte/macrophage-associated immunological disorders [88].

The iMph model was also used for HTS of antituberculosis drugs: by screening a library of 3,716 compounds, Han and co-authors [89] identified 120 hits, which led to the identification of a novel anti-*Mtb* compound, 10-DEBC.

Thus, the validity of the system for identifying drug candidates has been proven for several monocyte/macrophage-associated disorders.

## Modeling macrophage-pathogen interactions

Phagocytic activity and the production of microbicidal molecules are two characteristic macrophage features. iMph capacity to engulf pathogens and restrict their growth was demonstrated by several groups (Table 2).

**Table 2:** The use of iMphs to study macrophage-pathogen interactions

Infectious agent	Reference	Main findings
*Salmonella typhi,* *S. typhimurium*	Hale et al. [45]	iMphs are phagocytic and up-regulate inflammation-related genes in response to the infection
*P. aeruginosa*	Ackermann et al. [46]	Co-administration of *P. aeruginosa* and human iMphs to immunodeficient mice prevents the development of the infection; iMph administration shortly after the infection with *P. aeruginosa* (4 h) decreases infection severity
*S. aureus*	Hashtchin et al. [98]	Intratracheal injection of iMphs to immunodeficient humanized mice challenged with *S aureus* (incuding methicillin-resistant strain) reduces *S aureus* load, decreases granulocytic infiltration and diminishes lung pathology. Transcriptomic analysis: compared to MDMs, iMphs respond to the infection by a more profound upregulation of inflammatory genes early after infection, however the expression normalizes faster than in MDMs
*L. major*	O’Kneefe et al. [91]	After the infection, iMphs contain a higher level of intracellular *L. major* compared to THP-1, mouse and human bone marrow-derived macrophages
*M. tuberculosis*	Nenasheva et al. [33]	iMphs phagocyte and restrict *Mtb* growth in vitro
	Bernard et al. [90]	iMph infection with either virulent *Mtb* or the attenuated ESX-1-deficient *Mtb* mutant allowed to identify a role of ESX-1 secretion system of *Mtb* in the formation of autophagosomes and the subsequent *Mtb* escape from autophagosomes to the cytosol
	Haake et al. [47]	iMphs derived from patients with a complete or partial deficiency in IFN-γR2, IFN-γR1 or STAT1 demonstrate a defective upregulation of HLA-DR, CD64, CD38 and CD282 in response to IFNγ, a decreased phosphorylation of STAT1, no-to-little clearance of BCG. Additionally, STAT1-deficient iMphs have a disturbed production of ROS
	Han et al. [89]	iMphs are suitable to search for new anti-infectious drugs: the screening of a library of 3.716 compounds for their anti-*Mtb* activity was performed and a novel anti-*Mtb* compound, 10-DEBC, was identified
HIV-1	van Wilgenburg et al. [27]	iMphs are infectable with HIV-1
	Vaughan-Jackson et al. [92]	iMphs are infectable with HIV-1 and ZIKV
	Taylor et al. [96]	CRISPR/Cas9 engineered iMphs with depleted *USP18* exhibit: an increased reactivity to IFNI, a prolonged STAT1 and STAT2 phosphorylation; an enhanced expression of IFN‐stimulated genes; an increased restriction of HIV replication
ZIKV, DENV	Lang et al. [93]	Differences in iMph response to DENV and ZIKV have been demonstrated: DENV induced a higher inflammatory response, a higher production of MIF and a decreased iMph migration; ZIKV inhibited NF-kB signaling pathway

*DENV* dengue virus, *MIF* macrophage migration inhibitory factor, *ZIKV* zika virus

In in vitro analyses, iMphs (EB-S) efficiently phagocytosed *Salmonella typhi* and *S. typhimurium* and killed them more efficiently than THP-1 cells did [45]. In our study, iMphs (EB-S) effectively phagocytosed and restricted the growth of *Mycobacterium tuberculosis* (*Mtb*) [33]. By infecting iMphs with virulent wild-type *Mtb* and the attenuated *Mtb* mutant lacking the ESX-1 (early secreted antigen 6 kilodaltons system 1) secretion system, Bernard and co-authors [90] identified a role for the ESX-1 secretion system in the formation of autophagosomes and subsequent *Mtb* escape from autophagosomes to the cytosol.

Inborn errors of IFN-γ immunity underlie Mendelian susceptibility to mycobacterial disease. The iMph model has recently been used to study how inborn errors of IFN-γ immunity affect macrophage reactivity to IFN-γ and mycobacteria [31, 47]. iMphs were generated from patients with a complete or partial AR deficiency in the IFN-γ signaling pathway (i.e., a complete or partial deficiency in IFN-γR2, a partial deficiency in IFN-γR1 and a complete deficiency in STAT1). Following stimulation with IFN-γ, patient-derived iMphs exhibited defective upregulation of HLA-DR, CD64, CD38 and CD282, low to no degree of phosphorylation of STAT1 and no-to-little clearance of BCG. In addition, in iMphs derived from a STAT1-deficient patient, the generation of ROS was disturbed. Thus, the use of the iMph platform allowed to determine the pathways hampered in the macrophages of patients with inborn errors of IFN-γ immunity.

Differently from the high antibacterial activity of iMphs in relation to *S. typhimurium* and *Mtb*, iMphs appeared to be more permissive towards *Leishmania major* parasites: when infected with this pathogen, iMphs had a higher level of intracellular *Leishmania major* compared to THP-1, mouse and human bone marrow-derived macrophages [91].

iMph infectability with viruses was addressed in a few studies. van Wilgenburg and co-authors [27] and Vaughan-Jackson and co-authors [92] showed iMph infectability with HIV, although at a low rate [92]. Lang and co-authors [93] demonstrated that iMphs can be productively infected with Zika (ZIKV) and Dengue (DENV) viruses. The use of the iMph model allowed the authors to identify the differences in the inflammatory responses induced by ZIKV and DENV, particularly, a higher inflammatory response and a higher secretion of macrophage migration inhibitory factor (MIF), as well as a decreased migration of DENV-infected iMphs compared to ZIKV-infected ones and the inhibition of the NF-kB signaling pathway in ZIKV-infected iMphs. In a recent preprint study, iMphs were used to study the effects of human macrophages on SARS-CoV-2 (Severe acute respiratory syndrome-related coronavirus 2) infection. Using a co-culture system consisting of iMphs and iPSC-derived lung cells, the authors demonstrated that the inhibition of viral entry into the target cells using antibody blocking angiotensin-converting enzyme 2 (ACE2) enhanced the activity of M2 macrophages; the latter were able to eliminate SARS-CoV-2 without the induction of severe inflammatory responses and IL-6 and IL-18 overproduction [94].

Fundamentally, the possibility of generating homogeneous populations of iMphs in high quantities provides a unique opportunity to directly compare the reactivity of the same phagocyte population to a number of different pathogens, which is important to unravel intracellular mechanisms restricting/permitting different infections.

Another important and unique application of iMphs in the field of host–pathogen interactions is the identification of individual genes controlling macrophage functionality. The generation of iMphs and dendritic cells from iPSCs bearing biallelic mutations in the transcriptional factor IRF5 allowed to investigate the role of IRF5 in mediating the response of human myeloid cells to the influenza A virus and to demonstrate a reduction of the virus-induced inflammatory cytokine production under the conditions of IRF5 deficiency [95]. Taylor and co-authors [96] used iMphs to analyze the role of ubiquitin-specific proteinase 18 (USP18), a negative regulator of type I IFN signaling [97], in anti-viral response. The authors have demonstrated that: (i) infection of iMphs with HIV-1 induces USP18; (ii) depletion of USP18 with CRISPR/Cas9 increases iMph reactivity to IFNI, the phosphorylation of STAT1 and STAT2, the expression of IFN-stimulated genes and ultimately results in a significant restriction of HIV replication in iMphs. Thus, the use of the iMph model allowed to find the molecular target, modifying which it is possible to change the macrophage function in a desired way.

Finally, a prospective application of iMphs that has recently begun to be developed is a therapy of respiratory infections. Previously, Ackermann and co-authors demonstrated that human iMphs co-administered with *P. aeruginosa* to immunodeficient humanized mice prevented the development of *P. aeruginosa* infection; when injected shortly after the pathogen, iMphs rescued mice from severe infection [46]. More recently, the same group reported that intrapulmonary injection of human iMphs significantly reduced bacterial load and local pulmonary inflammation in immunodeficient mice challenged with *Staphilococcus aureus* 4 h prior to cell transfer [98]. Of note, iMphs responded to *S. aureus* infection by a profound upregulation of inflammatory genes soon after the infection, but quickly restored their stationary state, which is considered as a feature important for future iMph therapeutic use.

Overall, the iMph model is instrumental for the elucidation of genes/pathways involved in macrophage infectious control and may have prospects for future applications in the treatment of infectious diseases.

## Fundamental studies: elucidating the role of individual genes and factors in macrophage functionality and differentiation

The possibility of obtaining genetically edited iMphs allows to use the iMph model to perform fundamental analyses of how individual genes control macrophage activity.

Leucine-rich repeat kinase 2 (LRRK2) is a cytoplasmic multidomain protein containing GTPase, leucine-rich repeat and kinase domains. Mutations of the *LRRK2* gene are implicated in PD, predispose to Crohn’s disease and increase host susceptibility to intracellular pathogens [99]. How LRRK2 contributes to macrophage functionality is not fully clear, largely due to the lack of adequate human models. To explore the function of LRRK2 in human myeloid cells, Cowley’s group analyzed iMphs (EB-F) and iMG cells differentiated from PD patient-derived, control and edited iPSC lines. The authors reported that LRRK2 was not involved in the phagocytosis uptake of particles, but it was required for the recruitment of RAB8a and RAB10 proteins to phagosomes, and its expression was upregulated by IFN-γ. In another study, the generation of human iMphs (EB-F) with knockout of receptor-interacting serine/threonine-protein kinase 1 (*RIPK1)* allowed to identify a role for RIPK1 in the regulation of inflammatory and cell death pathways [100].

Apart from single gene analysis, the iMph model allows to use system approach to scrutinize genetic effects on macrophage functionality. In a proof-of-principle study, Navarro-Guerrero and co-authors [101] demonstrated the possibility of generating iMphs transduced with a screen of a CRISPR lentiviral library (a pool of 71,090 guides) and thus created a platform to screen the effects of a genome-wide loss-of-function knockout on macrophage characteristics.

In embryonic development, macrophages are formed during three different hematopoietic waves, i.e., primitive, pro-definitive and definitive. Primitive and pro-definitive waves take place in the yolk sac; at these stages, macrophages arise from primitive progenitors independently of hematopoietic stem cells (HSCs). During the definitive hematopoietic wave and adult hematopoiesis, macrophages differentiate from definitive HSCs in the bone marrow [102–104]. While human bone marrow samples are available for the analysis, the possibility of studying early embryonic hematopoietic processes in humans is ethically constrained. iMph differentiation provides a valuable model in this context. Indeed, both early embryonic hematopoiesis [105, 106] and iMph differentiation [34] are independent of the transcriptional factor c-Myb. During pro-definitive hematopoiesis, macrophages arise from erythromyeloid precursors [102, 103]; the formation of erythromyeloid precursors in iMph differentiation cultures has also been documented [41]. Finally, the primitive character of myelopoiesis occurring during human iMph differentiation has recently been confirmed using single-cell genomics [107]. Thus, the iMph model allows to look into the early processes of human myelopoiesis. Using this model, Ackermann and co-authors [108] were able to unravel a previously unknown role for IL-3 in the formation of hematopoietic progenitors and their myeloid specification. Some factors used for iMph generation are known to preferentially support either primitive (Activin A) or definitive (Wnt) hematopoiesis [109, 110]. Exploring how these factors, either together or separately, affect iMph differentiation trajectories, would help to achieve a better understanding of embryonic myelopoiesis.

Overall, the iMph model is valuable for studying the effects of individual genes and factors on early hematopoietic processes, myeloid specification and macrophage function.

## Developing macrophage-based cell therapy

The implication of macrophages in the pathogenesis of several hereditary diseases, their capacity to protect the host against infections and tumors, as well as their high immunoregulatory potential make macrophages an attractive tool for cell therapy of various diseases. Below we summarize the main areas of iMph-based cell therapy that are currently under development (summarized in Table 3) and discuss the advantages of the use of iMphs and MDMs for these purposes.

**Table 3:** The development of approaches for iMph-based cell therapy

Disease/ application	Reference	iPSC/iMph source	iPSC/iMph genetic modification (other manipulations)	Model	Main results
PAP	Lachmann et al. [115]	PAP patient (mutation in C*SF2RA* exon7)	Lentiviral transduction of CSF2RA transgene to PAP-iPSCs	In vitro analysis of PAP-iMphs and genetically corrected PAP-iMphs	PAP-iMphs: a reduced response to GM-CSF: an impaired CD11b upregulation, a decreased GM-CSF uptake, a hampared phagocytosis, a reduced STAT5-phosphorylation Corrected PAP-iMphs: correction of iMph response to GM-CSF
	Kuhn et al. [116]	PAP patient (mutation in C*SF2RA* exon7)	TALEN-mediated integration of *CSF2RA* into PAP-iPSCs;	In vitro analysis of PAP-iMphs and genetically corrected PAP-iMphs	Corrected iMphs: a restoration of cell response to GM-CSF: restored STAT5 phosphorylation and GM-CSF uptake
	Mucci et al. [117]	BL/6 (CD45.1) WT mice	-	intratracheal transplantation of WT iMphs into Csf2rb^−/−^ BL/6 (CD45.2) recipients	iMph therapeutic efficacy: a reduced opacity and protein levels in the BALF of recipient mice, an improved CT and lung tissue histology iMph biodistribution /persistence: iMph accumulate in alveolar spaces; iMph can be detected for up to 6 months
	Happle et al. [156]	Healthy donor	-	Weekly intratracheal transplantations of iMphs into humanized PAP mice (4 weeks)	iMph therapeutic efficacy: a reduced BALF protein level, a reduced level of surfactant D iMph biodistribution/safety: iMphs are found in the lungs near large airways, but not in other tissues (except human RNA been detected in the spleens of recipient mice); no signs of teratoma or tumors were recorded
ADA deficiency	Litvack et al. [114]	Mouse ESCs	- (iMph conditioning with GM-CSF and other factors to generate AL-iMphs)	Repeated intranasal administration to untreated ADA^−/−^ mouse pups; Single i.t. administration to 4 week-old ADA^−/−^ mice (previously treated with PEG-ADA)	iMph therapeutic efficacy: an increased mice survival in the absence of the other therapy; blood oxygen saturation was recovered; mucous substance in the alveoli was reduced; signs of pulmonary epithelial repair were detected
Infectious diseases	Ackermann et al. [46]	Healthy donor	-	In vivo: i.t. transfer into immunodeficient humanized mice infected with *P. aeruginosa* simultaneously with or 4 h prior to iMph transfer	iMph therapeutic efficacy: a reduction of infection scores, including a reduction of hemmorage, granulocytic infiltration of the lung tissue, edema and weight loss
	Taylor et al. [96]	Healthy donor	USP18 knock-out using CRISPR/Cas9	In vitro infection with HIV-1	In vitro effects: a reduced HIV-1 replication in engineered iMphs
Cancer	Senju et al. [23]	Healthy donor	iPSC electroporation with scFv specific to amyloid-β or CD20	In vitro: phagocytosis of amyloid-β-coated microbeads; engulfment and digestion of BALL-1 tumor cells; In vivo: simultaneous transfer of aCD20-iMphs and BALL-1 to SCID mice	Anti-amyloid-β-iMphs: an enhanced phagocytosis of amyloid-β coated microbeads; Anti-CD20-iMphs: the digestion of BALL-1 cell line in vitro; the inhibition of tumor growth in vivo
	Koba et al. [130] Senju et al. [131]	Healthy donor	iPSC electroporation with scFv specific to HER2/neu linked to FcgRI; lentiviral transduction of iPS-ML with *IFN-α, IFN-β, IFN-γ, TNF-α, FAS-ligand*, or *TRAIL*	In vitro*:* co-culture with human gastric (NUGC-4) and pancreatic (MIAPaCa-2) cancer cell lines In vivo*:* the transfer of PKH26-labeled iPS-MLs to SCID mice 15 days after the transfer of NUGC-4 or MIAPaCa-2 cell lines	In vitro effects*:* the inhibition of tumor growth (most efficient for HER2/neu-iMphs) Therapeutic efficacy: no anti-cancer activity of HER2/neu-iMphs; inhibition of tumor growth by iPS-ML/IFN-β and iPS-ML/anti-HER2/IFN-β
	Miyashita et al. [132]	Healthy donor	Lentiviral transduction of iPS-ML with *IFN-α* or *IFN-β*	In vitro: inhibition of human malignant melanoma cell line SK-MEL28 growth In vivo*:* intreperitoneal transfers of PKH26-labeled iPS-MLs to SCID mice bearing SK-MEL28 melanoma	In vitro effects*:* tumor growth inhibition Therapeutic efficacy: an inhibition of tumor growth by iPS-ML/IFNα, iPS-ML/IFNβ, and iPS-ML/IFNα + iPS-ML/IFNβ Biodistribution / safety: iMphs are found in the tumors; no signs of malignancy from human iPS-MLs at week 12 post-transfer
	Zhang et al. [133]	Healthy donor	Lentiviral transduction with anti-CD19 CAR and anti-mesothelin-CAR	In vivo*:* transplantation of CAR-Meso-iMphs activated in vitro by IFN-γ to NSG mice injected with ovarian cancer cells HO8_910_	Therapeutic efficacy: a reduction of tumor burden iMph persistence: CAR-iMphs persisted till more than 20 days and disappeared after day 30
Bone formation	Jeon et al. [151]	Healthy donor	-	In vitro: co-culture of iMphs with MSCs on scaffolds in osteogenic conditions; In vivo*:* s.c. transplantation of scaffolds seeded with MSCs and iMphs into nude mice	In vitro* & *in vivo effects: an acceleration of bone formation Safety: no teratoma formation was observed around the site of the implant at week 8 post-transplantation
Liver fibrosis	Pouyanfard et al. [150]	Healthy donor	-	In vivo*:* transplantation to immunodeficient mice with liver fibrosis	Therapeutic efficacy: a reduction of the expression of fibrinogenic genes and histological disease markers

*ADA* adenosine deaminase, *AL-iMphs* alveolar-like iMphs, *BALF* broncho-alveolar fluid, *BALL-1* B-cell leukemia cell line, *CAR* chimeric antigen receptor, *CSF2RA* colony stimulating factor 2 receptor, *CT* computed tomography, *iPS-ML* iPSC-derived myeloid/macrophage cell line, *MSC* mesenchymal stem cells, *PAP* pulmonary alveolar proteinosis, *PEG-ADA* polyethylene glycol–conjugated ADA, *WT* wild type, *scFv* single chain variable region fragment

### Correction of altered AM function

Perhaps the most developed direction of iMph-based cell therapy is that aimed at the correction of AMs for pulmonary disease treatment. Indeed, altered AM function underlies a variety of pulmonary diseases, including PAP, chronic obstructive disease, cystic fibrosis and adenosine deaminase deficiency (ADA). As shown recently, AMs represent a pool of macrophages that populate the lungs during embryogenesis; throughout a person's lifetime, the cells self-maintain locally and they are not at all or only weakly repopulated by MDMs [102, 105, 111–113]. Because the generation of iMphs recapitulates embryonic hematopoiesis [34], it has been suggested that in contrast to MDMs, iMphs could effectively replenish the AM pool [114]. The approach has been tested on several models.

In PAP model, several proof-of-principle studies demonstrated the possibility of efficiently correcting CSF2RA-deficiency and restoring human iMph functionality using lentiviral gene transfer. In these studies, iPSCs were obtained from CSF2RA deficient patients, genetically corrected and successfully differentiated into functional iMphs (OP9-dependent or EB-S protocols). The latter demonstrated restored phagocytic activity, GM-CSF uptake, intracellular signaling and surfactant clearance capacity [70, 115, 116].

The feasibility of correcting the AM pool in vivo, was proven in animal experiments. *Csfr2b*^−/−^ mice are considered as an experimental model of PAP. Following the intratracheal administration of wild-type mouse iMphs (EB-S) to *Csfr2b*^−/−^ recipients, donor cells migrated predominantly to the alveolar spaces, acquired AM-similar transcriptional signature, persisted in the lungs for as long as 2 months and improved PAP disease parameters [117]. The other PAP model utilizes ADA^−/−^ mice. In humans, ADA deficiency is an AR metabolic disorder that affects proliferating cells, causes immunodeficiency, predisposes to the development of PAP and manifests as pneumonia, chronic diarrhea, and widespread skin rashes. ADA^−/−^ mice die of respiratory failure within 18–21 days of birth. Litvack and co-authors [114] generated alveolar-like iMphs (EB-F) by conditioning mouse iMphs with GM-CSF and other factors. A single intratracheal administration of the resulting iAMs to 4-week-old ADA^−/−^ mice or repeated intranasal transfer of the cells to mouse pups significantly increased recipient survival, restored blood oxygen saturation and reduced mucous substance in the alveoli.

These experimental studies have demonstrated the prospects of using genetically corrected iMphs for cell therapy of macrophage-associated hereditary diseases, at least, pulmonary ones.

### Infectious disease treatment

Until recently, examinations of iMph-pathogen interactions have mainly been restricted to the analysis of iMph infectability and anti-infectious activity in vitro. However, one may expect the appearance and the exponential growth of studies aiming to increase iMph anti-infective potential and to use corrected iMphs for infectious disease treatment.

As a proof of principle, Lachmann’s group documented the anti-bacterial effect of unmodified human iMphs (EB-S) intratracheally administered to mice simultaneously with or shortly after their infection with *P. aerugenosa* or *S. aureus* [46, 98]. Taylor and co-authors [96] reported an increased restriction of HIV-1 replication by iMphs (EB-S) with depleted *USP18* gene (discussed above).

### Cancer cell therapy

In cancer, tumor microenvironment polarizes tumor-infiltrating macrophages to an anti-inflammatory state resulting in the generation of so-called tumor-activated macrophages (TAMs). TAMs exhibit diminished anti-tumor and increased tumor-promoting activities, which contrasts with M1-like macrophages that are anti-tumorigenic [118–120]. Because it is possible to “educate” M1-like macrophages in vitro and because macrophages have a high capacity to migrate to and infiltrate tumors [121], it was supposed that it would be possible to achieve an antitumor effect by transferring M1-like macrophages generated from blood monocytes and “educated” in vitro in the presence of IFN-γ or LPS [122]. Unfortunately, the approach was not effective. The underlying reasons are not fully clear, but may include a suboptimal number of transferred cells, insufficient anti-tumor activity of the educated macrophages and/or the instability of the M1 macrophage phenotype in tumor microenvironment (reviewed in [120]). To overcome the limitations, a suggestion was made to use genetically engineered macrophages that stably produce factors promoting immune activation (such as IL-12 or IFNI) [123] or express a chimeric antigen receptor (CAR) specific to tumor antigens (reviewed in [124, 125]). In several studies, CAR-bearing macrophages were successfully generated from J774A, THP1 macrophage-like cell lines and human primary macrophages using lentiviral or group B adenoviral (Ad5f35) vectors [126, 127]. The generated CAR-macrophages specifically recognized the cognate CD19, CD22 or HER2 antigens, phagocytosed antigen-bearing tumor cells, cross-presented tumor antigens, provided T-cell costimulation and inhibited tumor growth in vitro. Despite these first promising studies, the generation of genetically engineered macrophages from MDMs remains challenging, which is largely due to the restricted expansion ability of macrophages and their general resistance to genetic modifications [128, 129]. In this regard, iMphs may represent a more feasible way to develop “anti-tumor” macrophages.

In 2011, Senju and co-authors [23] generated human iMphs (OP9-dependent) expressing single chain variable region fragment (scFv) of antibodies specific to CD20 and demonstrated that the cells engulfed and killed B-cell leukemia cells. A series of later studies by the same group examined the potential of iPSC-derived myeloid cell lines (iPS-MLs) for restricting solid tumors. iPS-MLs are close but not identical to iMphs. The cells are generated by obtaining iPSC-derived myeloid cells and transducing them with genes that promote cell proliferation and limit cell senescence, such as c*MYC.* The resulting population represents actively proliferating myeloid cell lines that can easily be genetically manipulated and expanded [130, 131]. iPS-MLs (OP9-dependent) transduced with scFv specific to human HER2/neu antigen along with *IFNα, IFNβ, IFN-γ, TNF-α, TRAIL* or *FAS-ligand* genes were generated and their anti-tumor activity was tested in severe combined immunodeficiency (SCID) mice with human gastric and pancreatic cancer. In this model, all iPS-MLs accumulated in tumor tissues, but only iPS-MLs expressing IFN-β significantly inhibited tumor growth [130, 131]. In another study by the same group, iPS-MLs, genetically modified to express type I IFN, inhibited disseminated human melanoma in SCID mice [132].

Zhang and co-authors [133] generated human iMphs (EB-F) expressing CAR specific to CD19 (“CAR (CD19)-iMac”) or mesothelin (“CAR (meso)-iMac”). Detailed characterization of CAR-iMphs showed that the cells: (i) expressed surface markers and transcriptome characteristics for macrophages; (ii) were largely homogeneous (shown by single-cell RNA-sequencing); (iii) were initially biased towards the M2 state, but could be polarized towards M1 by in vitro treatment with IFN-γ; (iv) exerted antigen-specific anti-tumor effect in vitro and in vivo (the latter—following xeno-transplantation to NSG mice with ovarian cancer*).* Tracking CAR (meso)-iMacs following their transfer to immunodeficient NSG mice showed that the cells expanded during the first 3 days, persisted for about 20 days and disappeared after around day 30 post-transfer. This detailed study has provided a proof-of-principle for the utility of CAR-iMph-based cancer cell therapy and has also started monitoring iMph fate in vivo, which is important to understand the safety of iMph-based cell therapy (discussed below).

To conclude, genetic modification of iMphs so as to change their activity in a desired direction is a highly promising approach for anticancer cell therapy, and a rapid development of the field in the nearest future is expected.

### Inflammation control, wound healing and tissue regeneration

M2-like macrophages have potential clinical applications in the area of immune suppression, wound healing and tissue regeneration [134]. The effects of macrophages pre-treated in vitro with M2-polarizing agents, such as IL-4 combined with IL-13, IL-10 or TGF-β, hypo-osmotic shock or other stimuli, have been explored in different models in experimental and clinical settings. M2-like macrophages exerted an anti-inflammatory effect and alleviated the pathology of renal injury [135–137], spinal cord injury [138, 139] and sepsis-induced acute lung injury [140]; promoted angiogenesis [141] and stimulated the healing of pressure and diabetes wounds [142, 143]. However, the outcomes of M2 treatment were not fully reproducible (reviewed in [144]), which, again, was attributed to the functional instability of in vitro polarized macrophages and their return to a neutral or even proinflammatory phenotype in in vivo surrounding [145, 146].

iMphs were shown to be initially oriented towards an anti-inflammatory profile [33, 147], and to be responsive to M2-polarizing stimuli [148]. This potentially makes them well suited for immune regulation and tissue repair purposes. However, only a few studies have tested these iMph applications so far. In the study by Haideri and co-authors [149] mouse iMphs derived from embryonic stem cells reduced carbon tetrachloride-induced liver injury and fibrosis, diminished the number of activated myofibroblasts and activated liver progenitor cells. In a more recent study by Pouyanfard and co-authors [150], human iMphs (EB-S), preliminarily polarized towards the M2 subtype and transferred to immunodeficient mice with liver fibrosis, induced downregulation of the expression of fibrogenic genes and reduced disease histopathology. A different kind of iMph regenerative potential was demonstrated by Jeon and co-authors [151], who showed that co-cultivation of iMphs (EB-S) with iPSC-derived mesenchymal stem cells in a 3D culture model significantly improved bone formation in vitro and in vivo compared to a mono-culture of osteogenic cells. Other applications of iMph immune regulatory potential are yet to be tested, including in autoimmune and autoinflammatory diseases. In this regard, the generation of iMphs genetically engineered so as to maintain stable M2 activity looks promising.

## Macrophage-based cell therapy: iMphs *versus* MDMs

Given the general similarity of human iMphs and MDMs it is important to compare their potential as a tool for cell therapy (summarized in Table 4).

**Table 4:**
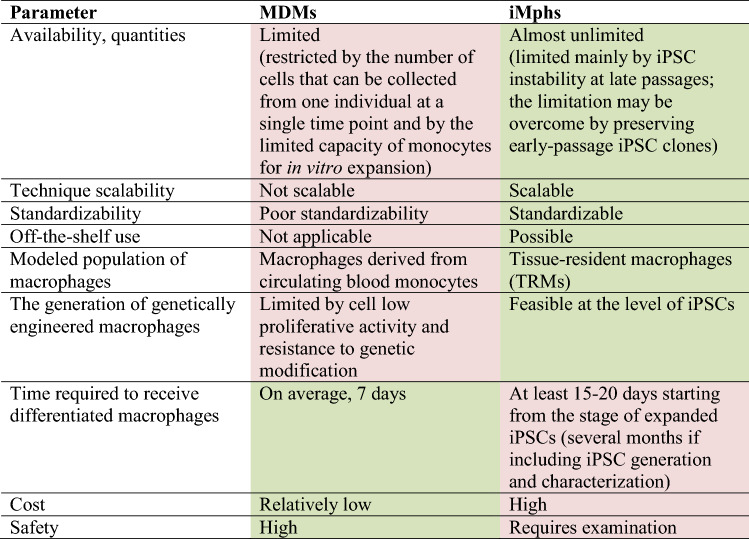
The benefits and limitations of MDM- and iMph-based cell therapy

Green, benefits; pink, limitations

iPSCs are easily and almost unlimitedly expandable. This creates the basis for: (i) obtaining iMphs in unlimited quantities from any individual; (ii) generating standardizable iMph populations. Moreover, techniques to scale up iMph production have recently been developed [35, 46]. Altogether, this creates the conditions for generating iMphs for “off-the-shelf” use.

In contrast to iMphs, MDMs can be obtained only in a limited quantity from a given individual, and their expansion in cultures is limited and temporal. Additionally, due to the restricted number of monocytes that can be collected from one individual at a single time point and the variability of monocyte populations collected at different time points even from the same individual, MDMs are much less standardizable.

Another advantage of iMphs over MDMs is the fact that the generation of iMphs recapitulates embryonic hematopoiesis and results in the formation of macrophages that reliably model TRMs [34, 41, 44]. Moreover, the differentiation of iMphs in tissue-specific conditions allows generating “tissue-specific” macrophages, e.g., cells recapitulating Kuppfer cells or microglia [20, 152, 153]. In contrast to iMphs, MDMs primarily model macrophages that repopulate tissues in inflammatory conditions [20].

An important benefit of iMphs is the ease of performing iPSC genetic modification and as a consequence, it becomes feasible to generate genetically engineered iMphs. Genetic modification of MDMs is much more challenging (although not fully impossible [126]) due to their low proliferative activity and natural resistance to genetic modification [128, 129, 134].

For their part, MDMs have their own benefits. First, the generation of MDMs is significantly cheaper compared to that of iMphs, as it requires only one exogenous factor (most often, M-CSF) and can be accomplished in 7 days [20]. This stands in contrast to the use of at least two or even up to nine exogenous factors for iMph generation and a much longer period required to generate iMphs (especially if considering the time frame needed for the generation of iPSCs; reviewed in [21]). Second, MDM administration has been shown to be safe and non-toxic [121, 153, 154]. The safety of iMph administration yet needs to be established.

## iMph-based cell therapy: safety and other questions to explore

When considering the prospects of iMph-based cell therapy, one must discuss iMph safety and other yet unresolved questions.

iMphs are terminally differentiated cells. However, they may have tumorigenic potential, primarily due to their potential contamination with residual iPSCs, which are immature cells with an almost unlimited proliferative activity. Furthermore, tumors may arise from iMph hematopoietic progenitors and due to genetic alterations arising in vitro during cell expansion [155]. Only a few studies have addressed iMph tumorigenicity so far. They reported a lack of teratoma formation in immunodeficient mice transplanted with human iMphs and examined approximately 2 months post-transfer [151, 156]. However, in most studies and over a longer time frame, the tumorigenic potential of iMphs has not been carefully studied. To reduce iPSC tumorigenicity, different methods of their elimination from the differentiated progeny have been suggested [157, 158], but only one of them has been approbated in an iMph model so far. Specifically, Lachmann’s group introduced iPSCs with iCas9 gene, which upon the addition of a chemical inducer of dimerization induces cell apoptosis [32]. In vitro, 98% of the engineered iPSCs and their iMph progeny could be eliminated by this method, which may be considered as an efficient killing. However, this degree of iPSC elimination may still be insufficient to warrant iMph clinical safety. In addition, in vivo validation of the approach is needed.

The second question, also related to iMph safety, is that of iMph biodistribution and persistence. This question has not been systematically addressed either. Happle and co-authors [156] reported that following an intratracheal application of human iMphs to humanized PAP mice, the transplanted cells accumulated in the lungs and could not be detected in the liver, bone marrow or spleen by flow cytometry analysis. However, real-time PCR did detect human RNA in the spleens, indicating that a small number of the transferred cells may spread throughout different tissues even after local (intrapulmonary) delivery. Another study reported that following an intra-peritoneal application of human CAR-iMphs to tumor-bearing mice, the cells gradually disappeared after day 30 [133]. Yet, thorough systematic analyses of iMph persistence, biodistribution and tumorigenicity have not been performed and are critically needed.

The safety of genetically engineered iMphs is a separate question to consider. It largely depends on the specificity of the introduced corrections (e.g., a proper selection of the target DNA site and the design of single guide RNA in the case of CRISPR/Cas technology), the choice of delivery method (e.g., vector integration ability) and the lack of off-target effects. The general drawbacks of existing genetic engineering techniques have been systemized in a series of comprehensive analyses [159–162] and are beyond the scope of this review. In relation to macrophages, it should be noted that the differentiation of iMphs from iPSCs goes through several stages, includes multiple rounds of cell division, takes at least 2–3 weeks to obtain the first iMph harvest and may last for many months afterwards. Therefore, the specificity and the stability of modifications introduced into iPSCs need to be additionally and carefully controlled at the iMph level. Furthermore, the phenotypes, transcriptomic signatures and functional activities of genetically modified iMphs should be carefully examined, as they may be influenced by the introduced mutations [57].

Potentially, for cell therapy purposes, allogeneic and autologous iMphs can be used. A significant benefit of allogeneic iMphs is their possible off-the-shelf use, which reduces therapy costs and the time it takes to prepare a cell product. However, allogeneic transplantation bears the risks of graft-derived infections and graft rejection. The first may be overcome by a thorough medical examination of the donor. The second problem may be solved by creating biobanks of iPSCs and iMphs with diverse HLA haplotypes [163, 164]. Another way to go, which is currently being actively elaborated, is to create “universal” iPSCs with ablated expression of HLA and/or other molecules involved in antigen presentation (e.g., TAP-1, CIITA) [155, 165, 166]. Such iPSCs can be used as a source for generating “universal” iMphs. To avoid the rejection of donor cells by the recipient’s innate immune cells, it has recently been suggested that immune regulatory molecules, such as CD47, could be introduced into HLA-ablated donor cells [167, 168]. However, it should be taken into account that decreased immunogenicity may lead to increased tumorigenicity. Therefore, the safety of hypoimmunogenic iMphs and their potential contamination with iPSCs should be examined with special attention, the benefit/risk ratio must be carefully evaluated, and the cells should be used only when critically needed. Moreover, for some applications, allogeneic iMphs may have preferences over the “universal” ones. For example, if iMphs are intended to eradicate an acute infection, a short-term survival of donor cells may be preferred, as this will reduce the tumorigenicity risk [155].

Autologous iMphs are devoid of infection and rejection flaws. However, their generation is expensive and time-consuming. As a result, they can be used for the treatment of chronic diseases only. Additionally, iPSC lines derived from different individuals may differ, which is why their safety and efficacy may also vary [155]. Finally, the tumorigenicity risk may be higher for autologous iPSC-derived cells compared to allogeneic cells due to a low immunogenicity of the former [169, 170].

Overall, allogeneic and autologous iMphs have their own advantages and limitations; the choice may depend on the cost-efficacy ratio and on the application the cells are intended for, particularly, on whether they are intended for the treatment of acute or chronic conditions.

Apart from the safety issues, there are other questions that need to be solved before iMph therapy becomes a reality. Technically, we need to optimize iMph differentiation methodologies so as to increase iMph output, to decrease iMph generation cost and to warrant differentiation stability, iMph homogeneity and purity. The use of genetically modified iMphs requires further improvement of reprogramming methods [159]. In contrast to embryonic stem cells, iPSC generation does not require the destruction of human embryos. Nonetheless, ethical issues still remain, including the risk of tumor generation in the process of stem therapy, donor selection in the case of allogeneic transplantation, and the enforcement of restrictions not to use iPSCs to make human embryos, produce human germ cells and clone human beings. Finally, a regulatory path for the use of iPSC derived cell products needs to be developed. These issues have been reviewed elsewhere [159, 171] and are beyond the scope of the present review.

## Conclusions

The generation of macrophages from iPSCs is a recently developed technique that has several important advantages promoting a widespread use of the iMph model. The main iMph benefits include:

(i) The possibility of obtaining iPSCs/iMphs nearly from any donor and of any genetic background (availability) [35, 43, 166];
(ii) Potential feasibility of obtaining iMphs in unlimited quantities, including large-scale production in industry-compatible bioreactors (scalability) [35, 46];
(iii) Genetic, phenotypic, transcriptomic and functional homogeneity and a “naïve-like” state of the iMph population (standardizability) [33, 133];
(iv) The ease of obtaining genetically edited iMphs due to the feasibility of genetic modification of iPSCs (editability) [62, 115, 116, 159].

**Fig. 2:**
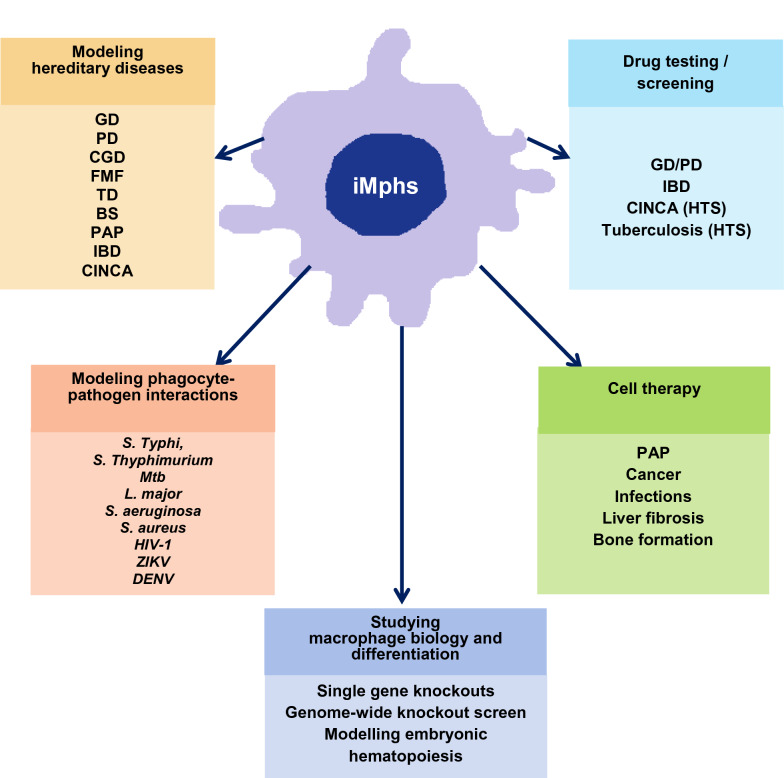
Main current and prospective iMph applications. Since the development of iMph generation techniques, the spectrum of iMphs applications has rapidly grown. Starting with the modeling of hereditary diseases associated with impaired macrophage function, it currently also includes the modeling of macrophage-pathogen interactions, drug testing and screening and the development of approaches to iMph-based cell therapy. *BS* Blau syndrome, *CGD* chronic granulomatous disease, *CINCA* chronic infantile neurologic cutaneous and articular syndrome; *DENV* Dengue virus, *FMF* familial Mediterranean fever, *GD* Gaucher disease, *HTS* high throughput screening, *IBD* inflammatory bowel disease, *Mtb* *Mycobacterium tuberculosis*, *PAP* pulmonary alveolar proteinosis, *PD* Parkinson’s disease, *TD* Tangier disease, *ZIKV* Zika virus

The advantages of the iMph model along with the multifaceted activity of the macrophage population in general underlie multiple applications wherein iMphs are valuable (Fig. 2). The main of them are:

(i) Disease modeling. The existing studies have focused mostly on the modeling of rare hereditary diseases. However, given the role of macrophages in the pathogenesis of many other disorders, it is anticipated that the spectrum of iMph-based disease models will be extended beyond rare diseases to include such pathologies as autoinflammatory disorders, atherosclerosis and others.
(ii) Studying macrophage-pathogen interactions. iMph infectability with various pathogens has been documented and used to study the molecular basis of macrophage-pathogen interactions. Future developments will likely include the elaboration of approaches to infectious disease treatment based on the use of “native” [46, 96, 98] or genetically engineered [96] iMphs.
(iii) Drug screening. In this area, the HTS of large compound libraries using the iMph model is especially promising.
(iv) In the area of basic research, the iMph platform allows to model embryonic myelopoiesis and to directly assess the effects of individual genes on macrophage functionality.
(v) Finally, an attractive direction is the use of iMphs for cell-based therapy. Potential applications include the treatment of hereditary diseases, adjunctive therapy for acute infections, inflammatory conditions and cancer, as well as replacement cell therapy. For most of these applications, our ability to modify iMphs so as to change their activity in a desired direction will be important.

In spite of the prospects of the iMph methodology, it is not without limitations. To make iMph clinical use a reality, multiple methodological, ethical and regulatory issues need to be solved. Among them, the safety of iMph use comes first. This requires filling research gaps in terms of iMph biodistribution, persistence and tumorigenicity. Further methodological studies are needed to increase iMph yield and differentiation stability, secure iMph purity and homogeneity and decrease iMph generation cost. For genetically manipulated iMphs, optimization of reprogramming techniques and strict control of cell stability, as well as the minimization of off-target effects are needed. Some biological factors, such as the differentiation potential of different iPSC lines, are difficult to predict. Therefore, reliable methods to assay the whole differentiation process and the resulting iMphs need to be elaborated and implemented. Finally, a regulatory path for (or against) the use of iPSC-derived cell products needs to be elaborated.

In conclusion, iMphs have multiple prospective applications, and their number is rapidly expanding. At the same time, the safety of using iMphs remains insufficiently researched. A thorough examination of this issue is an essential step towards a future clinical implementation of iMph-based therapy.

## Data Availability

Data sharing is not applicable to this article as no datasets were generated or analysed during the current study.

## Abbreviations

AM: Alveolar macrophages
AD: Alzheimer’s disease
ADA: Adenosine deaminase
AR: Autosomal recessive
BCG: Bacillus Calmette–Guérin
BMP4: Bone morphogenetic protein 4
BS: Blau syndrome
CAR: Chimeric antigen receptor
CGD: Chronic granulomatous disease
CINCA: Chronic infantile neurologic cutaneous and articular syndrome
CRISPR: Clustered regularly interspaced short palindromic repeats
CSF2R: Colony stimulating factor 2 receptor
DENV: Dengue virus
EB: Embryoid body
EB-S: Spontaneous EB-based protocol of iMph differentiation
EB-F: Factor-assisted EB-based protocol of iMph differentiation
FGF2: Basic fibroblast growth factor
FLT3L: FMS-like tyrosine kinase 3 ligand
FMF: Familial Mediterranean fever
GBA1: β-Glucocerebrosidase
GD: Gaucher disease
GM-CSF: Granulocyte–macrophage colony-stimulating factor
HE: Hemogenic endothelium
HIV: Human immunodeficiency virus
HPP: Hematopoietic progenitor
HSC: Hematopoietic stem cell
HTS: High throughput screening
IBD: Inflammatory bowel disease
IL-3: Interleukin-3
IL-6: Interleukin-6
iMG: iPSC-derived microglia
iMphs: iPSC-derived macrophages
iPSCs: Induced pluripotent stem cells
iPS-MLs: iPSC-derived myeloid cell lines
LPS: Lipopolysaccharide
LRRK2: Leucine-rich repeat kinase 2 (LRRK2)
M-CSF: Macrophage colony-stimulating factor
MDM: Monocyte-derived macrophages
Mtb: Mycobacterium tuberculosis
NLRP3: NOD-, LRR- and pyrin domain-containing protein 3
NOD2: Nucleotide-binding oligomerization domain 2
PAP: Pulmonary alveolar proteinosis
PD: Parkinson’s disease
RBCs: Red blood cells
ROS: Reactive oxygen species (ROS)
scFv: Single chain variable region fragment
SCF: Stem cell factor
SCID: Severe combined immunodeficiency
SNCA: α-Synuclein
TD: Tangier disease
TPO: Thrombopoietin
TREM2: Triggering Receptor Expressed On Myeloid Cells 2
USP18: Ubiquitin-specific proteinase 18
VEGFA: Vascular endothelial growth factor A
ZIKV: Zika virus
2D-F: 2D factor-dependent protocol of iMph differentiation

## References

[1] Takahashi K, Yamanaka S (2006). Induction of pluripotent stem cells from mouse embryonic and adult fibroblast cultures by defined factors. Cell.

[2] Mantovani A, Biswas SK, Galdiero MR, Sica A, Locati M (2013). Macrophage plasticity and polarization in tissue repair and remodelling. J Pathol.

[3] Wynn T, Chawla A, Pollard J (2013). Macrophage biology in development, homeostasis and disease. Nature.

[4] Weiss G, Schaible UE (2015). Macrophage defense mechanisms against intracellular bacteria. Immunol Rev.

[5] Watanabe S, Alexander M, Misharin AV, Budinger GRS (2019). The role of macrophages in the resolution of inflammation. J Clin Invest.

[6] Mosser DM, Edwards JP (2008). Exploring the full spectrum of macrophage activation. Nat Rev Immunol.

[7] Martinez FO, Gordon S (2014). The M1 and M2 paradigm of macrophage activation: time for reassessment. F1000prime Rep..

[8] Murray PJ, Allen JE, Biswas SK, Fisher EA, Gilroy DW, Goerdt S (2014). Macrophage activation and polarization: nomenclature and experimental guidelines. Immunity.

[9] Wynn TA, Vannella KM (2016). Macrophages in tissue repair, regeneration, and fibrosis immunity. Immunity.

[10] van Dalen FJ, van Stevendaal MHME, Fennemann FL, Verdoes M, Ilina O (2018). Molecular repolarisation of tumour-associated macrophages. Molecules.

[11] Atri C, Guerfali FZ, Laouini D (2018). Role of human macrophage polarization in inflammation during infectious diseases. Int J Mol Sci.

[12] Orekhov AN, Orekhova VA, Nikiforov NG, Myasoedova VA, Grechko AV, Romanenko EB (2019). Monocyte differentiation and macrophage polarization. Vessel Plus..

[13] Byrne AJ, Mathie SA, Gregory LG, Lloyd CM (2015). Pulmonary macrophages: key players in the innate defense of the airways. Thorax.

[14] Cassetta L, Pollard JW (2018). Targeting macrophages: therapeutic approaches in cancer. Nat Rev Drug Discov.

[15] Parisi L, Gini E, Baci D, Tremolati M, Fanuli M, Bassani B (2018). Macrophage polarization in chronic inflammatory diseases: killers or builders?. J Immunol Res.

[16] Guerrini V, Gennaro ML (2019). Foam cells: one size doesn't fit all. Trends Immunol.

[17] Galloway DA, Phillips AEM, Owen DRJ, Moore CS (2019). Phagocytosis in the brain: homeostasis and disease. Front Immunol.

[18] Merad M, Martin JC (2020). Pathological inflammation in patients with COVID-19: a key role for monocytes and macrophages. Nat Rev Immunol.

[19] Zhang C, Yang M, Ericsson AC (2021). Function of macrophages in disease: current understanding on molecular mechanisms. Front Immunol.

[20] Luque-Martin R, Mander PK, Leenen PJM, Winther MPJ (2021). Classic and new mediators for in vitro modelling of human macrophages. J Leukoc Biol.

[21] Lyadova I, Gerasimova T, Nenasheva T (2021). Macrophages derived from human induced pluripotent stem cells: the diversity of protocols, future prospects, and outstanding questions. Front Cell Dev Biol.

[22] Choi KD, Yu J, Smuga-Otto K, Salvagiotto G, Rehrauer W, Vodyanik M (2009). Hematopoietic and endothelial differentiation of human induced pluripotent stem cells. Stem Cells.

[23] Senju S, Haruta M, Matsumura K, Matsunaga Y, Fukushima S, Ikeda T (2011). Generation of dendritic cells and macrophages from human induced pluripotent stem cells aiming at cell therapy. Gene Ther.

[24] Kambal A, Mitchell G, Cary W, Gruenloh W, Jung Y, Kalomoiris S (2011). Generation of HIV-1 resistant and functional macrophages from hematopoietic stem cell-derived induced pluripotent stem cells. Mol Ther.

[25] Brault J, Goutagny E, Telugu N, Shao K, Baquié M, Satre V (2014). Optimized generation of functional neutrophils and macrophages from patient-specific induced pluripotent stem cells: ex vivo models of X(0)-linked, AR22(0)—and AR47(0)—chronic granulomatous diseases. Biores Open Access.

[26] Panicker LM, Miller D, Park TS, Patel B, Azevedo JL, Awad O (2012). Induced pluripotent stem cell model recapitulates pathologic hallmarks of Gaucher disease. Proc Natl Acad Sci USA.

[27] van Wilgenburg B, Browne C, Vowles J, Cowley SA (2013). Efficient, long term production of monocyte-derived macrophages from human pluripotent stem cells under partly-defined and fully-defined conditions. PLoS ONE.

[28] Alasoo K, Martinez FO, Hale C, Gordon S, Powrie F, Dougan G (2015). Transcriptional profiling of macrophages derived from monocytes and iPS cells identifies a conserved response to LPS and novel alternative transcription. Sci Rep.

[29] Lachmann N, Ackermann M, Frenzel E, Liebhaber S, Brennig S, Happle C (2015). Large-scale hematopoietic differentiation of human induced pluripotent stem cells provides granulocytes or macrophages for cell replacement therapies. Stem Cell Rep.

[30] Mukherjee C, Hale C, Mukhopadhyay S (2018). A simple multistep protocol for differentiating human induced pluripotent stem cells into functional macrophages. Meth Mol Biol.

[31] Neehus AL, Lam J, Haake K, Merkert S, Schmidt N, Mucci A (2018). Impaired IFNγ-signaling and mycobacterial clearance in IFNγR1-deficient human iPSC-derived macrophages. Stem Cell Rep.

[32] Lipus A, Janosz E, Ackermann M, Hetzel M, Dahlke J, Buchegger T (2020). Targeted integration of inducible caspase-9 in human iPSCs allows efficient in vitro clearance of iPSCs and iPSC-macrophages. Int J Mol Sci.

[33] Nenasheva T, Gerasimova T, Serdyuk Y, Grigor'eva E, Kosmiadi G, Nikolaev A (2020). Macrophages derived from human induced pluripotent stem cells are low-activated "naïve-like" cells capable of restricting mycobacteria growth. Front Immunol.

[34] Buchrieser J, James W, Moore MD (2017). Human induced pluripotent stem cell-derived macrophages share ontogeny with MYB-independent tissue-resident macrophages. Stem cell Rep.

[35] Gutbier S, Wanke F, Dahm N, Rümmelin A, Zimmermann S, Christensen K (2020). Large-scale production of human iPSC-derived macrophages for drug screening. Int J Mol Sci.

[36] Lopez-Yrigoyen M, May A, Ventura T, Taylor H, Fidanza A, Cassetta L (2020). Production and characterization of human macrophages from pluripotent stem cells. J Vis Exp.

[37] Zhang H, Xue C, Shah R, Bermingham K, Hinkle CC, Li W (2015). Functional analysis and transcriptomic profiling of iPSC-derived macrophages and their application in modeling Mendelian disease. Circ Res.

[38] Joshi K, Elso C, Motazedian A, Labonne T, Schiesser JV, Cameron F (2019). Induced pluripotent stem cell macrophages present antigen to proinsulin-specific T cell receptors from donor-matched islet-infiltrating T cells in type 1 diabetes. Diabetologia.

[39] Shi J, Xue C, Liu W, Zhang H (2019). Differentiation of human-induced pluripotent stem cells to macrophages for disease modeling and functional genomics. Curr Protoc Stem Cell Biol.

[40] Yanagimachi MD, Niwa A, Tanaka T, Honda-Ozaki F, Nishimoto S, Murata Y (2013). Robust and highly-efficient differentiation of functional monocytic cells from human pluripotent stem cells under serum- and feeder cell-free conditions. PLoS ONE.

[41] Takata K, Kozaki T, Lee C, Thion MS, Otsuka M, Lim S (2017). Induced-pluripotent-stem-cell-derived primitive macrophages provide a platform for modeling tissue-resident macrophage differentiation and function. Immunity.

[42] Cao X, Yakala GK, van den Hil FE, Cochrane A, Mummery CL, Orlova VV (2019). Differentiation and functional comparison of monocytes and macrophages from hiPSCs with peripheral blood derivatives. Stem cell Rep.

[43] Cui D, Franz A, Fillon SA, Jannetti L, Isambert T, Fundel-Clemens K, et al. High-Yield human induced pluripotent stem cell-derived monocytes and macrophages are functionally comparable with primary cells. Front Cell Dev Biol 2021; 9: 656867. 10.3389/fcell.2021.656867 10.3389/fcell.2021.656867 PMC8080307 33937256

[44] Lee C, Kozaki T, Ginhoux F (2018). Studying tissue macrophages in vitro: are iPSC-derived cells the answer?. Nature Rev Immunol.

[45] Hale C, Yeung A, Goulding D, Pickard D, Alasoo K, Powrie F (2015). Induced pluripotent stem cell derived macrophages as a cellular system to study salmonella and other pathogens. PLoS ONE.

[46] Ackermann M, Kempf H, Hetzel M, Hesse C, Hashtchin AR, Brinkert K (2018). Bioreactor-based mass production of human iPSC-derived macrophages enables immunotherapies against bacterial airway infections. Nature Comm.

[47] Haake K, Neehus AL, Buchegger T, Kühnel MP, Blank P, Philipp F (2020). Patient iPSC-derived macrophages to study inborn errors of the IFN-γ responsive pathway. Cells.

[48] Mistry PK, Liu J, Yang M, Nottoli T, McGrath J, Jain D (2010). Glucocerebrosidase gene-deficient mouse recapitulates Gaucher disease displaying cellular and molecular dysregulation beyond the macrophage. Proc Natl Acad Sci USA.

[49] Stirnemann J, Belmatoug N, Camou F, Serratrice C, Froissart R, Caillaud C (2017). A review of Gaucher disease pathophysiology, clinical presentation and treatments. Int J Mol Sci.

[50] Brady RO (2006). Enzyme replacement for lysosomal diseases. Annu Rev Med.

[51] Panicker LM, Miller D, Awad O, Bose V, Lun Y, Park TS (2014). Gaucher iPSC-derived macrophages produce elevated levels of inflammatory mediators and serve as a new platform for therapeutic development. Stem Cells.

[52] Messelodi D, Bertuccio SN, Indio V, Strocchi S, Taddia A, Serravalle S (2021). iPSC-Derived gaucher macrophages display growth impairment and activation of inflammation-related cell death. Cells.

[53] Xu L, Pu J (2016). Alpha-synuclein in Parkinson's disease: from pathogenetic dysfunction to potential clinical application. Parkinsons Dis.

[54] Haenseler W, Sansom SN, Buchrieser J, Newey SE, Moore CS, Nicholls FJ (2017). A highly efficient human pluripotent stem cell microglia model displays a neuronal-co-culture-specific expression profile and inflammatory response. Stem Cell Reports.

[55] Sidransky E, Nalls MA, Aasly JO, Aharon-Peretz J, Annesi G, Barbosa ER (2009). Multicenter analysis of glucocerebrosidase mutations in Parkinson’s disease. N Engl J Med.

[56] Aflaki E, Borger DK, Moaven N, Stubblefield BK, Rogers SA, Patnaik S (2016). A new glucocerebrosidase chaperone reduces α-synuclein and glycolipid levels in iPSC-derived dopaminergic neurons from patients with gaucher disease and parkinsonism. J Neurosci.

[57] Munn C, Burton S, Dickerson S, Bakshy K, Strouse A, Rajesh D (2021). Generation of cryopreserved macrophages from normal and genetically engineered human pluripotent stem cells for disease modelling. PLoS ONE.

[58] Segal BH, Veys P, Malech H, Cowan MJ (2011). Chronic granulomatous disease: lessons from a rare disorder. Biol Blood Marrow Transplant.

[59] Leiding JW, Holland SM. Chronic granulomatous disease. In: Adam MP, Ardinger HH, Pagon RA et al. GeneReviews^®^ [Internet]. 2012. University of Washington, Seattle; 1993–2021. https://www.ncbi.nlm.nih.gov/books/NBK99496/22876374

[60] Jiang Y, Cowley SA, Siler U, Melguizo D, Tilgner K, Browne K (2012). Derivation and functional analysis of patient-specific induced pluripotent stem cells as an in vitro model of chronic granulomatous disease. Stem Cells.

[61] Flynn R, Grundmann A, Renz P, Hänseler W, James WS, Cowley SA, Moore MD (2015). CRISPR-mediated genotypic and phenotypic correction of a chronic granulomatous disease mutation in human iPS cells. Exp Hematol.

[62] Klatt D, Cheng E, Philipp F, Selich A, Dahlke J, Schmidt RE (2019). Targeted repair of p47-CGD in iPSCs by CRISPR/Cas9: functional correction without cleavage in the highly homologous pseudogenes. Stem Cell Rep.

[63] Rust S, Rosier M, Funke H, Real J, Amoura Z, Piette JC (1999). Tangier disease is caused by mutations in the gene encoding ATP-binding cassette transporter 1. Nat Genet.

[64] Yvan-Charvet L, Ranalletta M, Wang N, Han S, Terasaka N, Li R (2007). Combined deficiency of ABCA1 and ABCG1 promotes foam cell accumulation and accelerates atherosclerosis in mice. J Clin Invest.

[65] Groenen AG, Halmos B, Tall AR, Westerterp M (2021). Cholesterol efflux pathways, inflammation, and atherosclerosis. Crit Rev Biochem Mol Biol.

[66] Gupta RM, Meissner TB, Cowan CA, Musunuru K (2016). Genome-edited human pluripotent stem cell-derived macrophages as a model of reverse cholesterol transport-brief report. Arterioscler Thromb Vasc Biol.

[67] Wouters CH, Maes A, Foley KP, Bertin J, Rose CD (2014). Blau syndrome, the prototypic auto-inflammatory granulomatous disease. Pediatr Rheumatol Online J.

[68] Takada S, Kambe N, Kawasaki Y, Niwa A, Honda-Ozaki F, Kobayashi K (2018). Pluripotent stem cell models of Blau syndrome reveal an IFN-γ-dependent inflammatory response in macrophages. J Allergy Clin Immunol.

[69] Carrington JM, Hershberger DM. Pulmonary alveolar proteinosis. In: StatPearls [Internet]. Treasure Island (FL). 2021. https://www.ncbi.nlm.nih.gov/books/NBK482308/29493933

[70] Suzuki T, Mayhew C, Sallese A, Chalk C, Carey BC, Malik P (2014). Use of induced pluripotent stem cells to recapitulate pulmonary alveolar proteinosis pathogenesis. Am J Respir Crit Care Med.

[71] Mukhopadhyay S, Heinz E, Porreca I, Alasoo K, Yeung A, Yang HT (2020). Loss of IL-10 signaling in macrophages limits bacterial killing driven by prostaglandin E2. J Exp Med.

[72] Hoffmann D, Sens J, Brennig S, Brand D, Philipp F, Vollmer Barbosa P (2021). Genetic correction of IL-10RB deficiency reconstitutes anti-inflammatory regulation in iPSC-derived macrophages. J Pers Med.

[73] Sens J, Hoffmann D, Lange L, Vollmer Barbosa P, Morgan MA, Falk CS, Schambach A (2021). Knock-out iPSCs for disease and therapy modeling of IL-10 associated primary immunodeficiencies. Hum Gene Ther.

[74] Finetti M, Omenetti A, Federici S, Roberta C, Marco G (2016). Chronic Infantile Neurological Cutaneous and Articular (CINCA) syndrome: a review. Orphanet J Rare Dis.

[75] Tanaka T, Takahashi K, Yamane M, Tomida S, Nakamura S, Oshima K (2012). Induced pluripotent stem cells from CINCA syndrome patients as a model for dissecting somatic mosaicism and drug discovery. Blood.

[76] McQuade A, Blurton-Jones M (2019). Microglia in Alzheimer's disease: exploring how genetics and phenotype influence risk. J Mol Biol.

[77] Katsumoto A, Takeuchi H, Takahashi K, Tanaka F (2018). Microglia in Alzheimer's disease: risk factors and inflammation. Front Neurol.

[78] Li Q, Barres B (2018). Microglia and macrophages in brain homeostasis and disease. Nat Rev Immunol.

[79] Colonna M, Wang Y (2016). TREM2 variants: new keys to decipher Alzheimer disease pathogenesis. Nat Rev Neurosci.

[80] McQuade A, Kang YJ, Hasselmann J, Jairaman A, Sotelo A, Coburn M (2020). Gene expression and functional deficits underlie TREM2-knockout microglia responses in human models of Alzheimer's disease. Nat Commun.

[81] Garcia-Reitboeck P, Phillips A, Piers TM, Villegas-Llerena C, Butler M, Mallach A (2018). Human induced pluripotent stem cell-derived microglia-like cells harboring TREM2 missense mutations show specific deficits in phagocytosis. Cell Rep.

[82] Piers TM, Cosker K, Mallach A, Johnson GT, Guerreiro R, Hardy J, Pocock JM (2020). A locked immunometabolic switch underlies TREM2 R47H loss of function in human iPSC-derived microglia. FASEB J.

[83] Cosker K, Mallach A, Limaye J, Piers TM, Staddon J, Neame SJ (2021). Microglial signalling pathway deficits associated with the patient derived R47H TREM2 variants linked to AD indicate inability to activate inflammasome. Sci Rep.

[84] Reich M, Paris I, Ebeling M, Dahm N, Schweitzer C, Reinhardt D (2021). Alzheimer’s risk gene TREM2 determines functional properties of new type of human iPSC-derived microglia. Front Immunol.

[85] Hall-Roberts H, Agarwal D, Obst J, Smith TB, Monzón-Sandoval J, Di Daniel E (2020). TREM2 Alzheimer's variant R47H causes similar transcriptional dysregulation to knockout, yet only subtle functional phenotypes in human iPSC-derived macrophages. Alzheimers Res Ther.

[86] Brownjohn PW, Smith J, Solanki R, Lohmann E, Houlden H, Hardy J (2018). Functional Studies of Missense TREM2 mutations in human stem cell-derived microglia. Stem Cell Reports.

[87] Han TU, Sam R, Sidransky E (2020). Small molecule chaperones for the treatment of Gaucher disease and GBA1-associated Parkinson disease. Front Cell Dev Biol.

[88] Seki R, Ohta A, Niwa A, Sugimine Y, Naito H, Nakahata T (2020). Induced pluripotent stem cell-derived monocytic cell lines from a NOMID patient serve as a screening platform for modulating NLRP3 inflammasome activity. PLoS ONE.

[89] Han HW, Seo HH, Jo HY, Han HJ, Falcão VCA, Delorme V (2019). Drug discovery platform targeting *M.tuberculosis* with human embryonic stem cell-derived macrophages. Stem Cell Rep..

[90] Bernard EM, Fearns A, Bussi C, Santucci P, Peddie CJ, Lai RJ (2020). *M. tuberculosis* infection of human iPSC-derived macrophages reveals complex membrane dynamics during xenophagy evasion. J Cell Sci.

[91] O'Keeffe A, Hale C, Cotton JA, Yardley V, Gupta K, Ananthanarayanan A (2020). Novel 2D and 3D assays to determine the activity of anti-leishmanial drugs. Microorganisms.

[92] Vaughan-Jackson A, Stodolak S, Ebrahimi KH, Browne C, Reardon PK, Pires E (2021). Differentiation of human induced pluripotent stem cells to authentic macrophages using a defined, serum-free, open-source medium. Stem Cell Rep.

[93] Lang J, Cheng Y, Rolfe A, Hammack C, Vera D, Kyle K (2018). An hPSC-derived tissue-resident macrophage model reveals differential responses of macrophages to ZIKV and DENV infection. Stem Cell Rep.

[94] Duan F, Guo L, Yang L, Han Y, Thakur A, Nilsson-Payant BE (2020). Modeling COVID-19 with human pluripotent stem cell-derived cells reveals synergistic effects of anti-inflammatory macrophages with ACE2 inhibition against SARS-CoV-2. Res Sq..

[95] Forbester JL, Clement M, Wellington D, Yeung A, Dimonte S, Marsden M (2020). IRF5 promotes influenza virus-induced inflammatory responses in human induced pluripotent stem cell-derived myeloid cells and murine models. J Virol.

[96] Taylor JP, Cash MN, Santostefano KE, Nakanishi M, Terada N, Wallet MA (2018). CRISPR/Cas9 knockout of USP18 enhances type I IFN responsiveness and restricts HIV-1 infection in macrophages. J Leukoc Biol.

[97] Basters A, Knobeloch KP, Fritz G (2018). USP18—a multifunctional component in the interferon response. Biosci Rep.

[98] Hashtchin AR, Fehlhaber B, Hetzel M, Manstein F, Stalp JL, Glage S (2021). Human iPSC-derived macrophages for efficient *Staphylococcus aureus* clearance in a murine pulmonary infection model. Blood Adv.

[99] Lee H, Flynn R, Sharma I, Haberman E, Carling PJ, Nicholls FJ (2020). LRRK2 is recruited to phagosomes and co-recruits RAB8 and RAB10 in human pluripotent stem cell-derived macrophages. Stem Cell Rep.

[100] Buchrieser J, Oliva-Martin MJ, Moore MD, Long JCD, Cowley SA, Perez-Simón JA (2018). RIPK1 is a critical modulator of both tonic and TLR-responsive inflammatory and cell death pathways in human macrophage differentiation. Cell Death Dis.

[101] Navarro-Guerrero E, Tay C, Whalley JP, Cowley SA, Davies B, Knight JC, Ebner D (2021). Genome-wide CRISPR/Cas9-knockout in human induced pluripotent stem cell (iPSC)-derived macrophages. Sci Rep.

[102] Hoeffel G, Chen J, Lavin Y, Low D, Almeida FF, See P (2015). C-Myb(+) erythro-myeloid progenitor-derived fetal monocytes give rise to adult tissue-resident macrophages. Immunity.

[103] Lacaud G, Kouskoff V (2017). Hemangioblast, hemogenic endothelium, and primitive versus definitive hematopoiesis. Exp Hematol.

[104] Hadland B, Yoshimoto M (2018). Many layers of embryonic hematopoiesis: new insights into B-cell ontogeny and the origin of hematopoietic stem cells. Exp Hematol.

[105] Schulz C, Gomez Perdiguero E, Chorro L, Szabo-Rogers H, Cagnard N, Kierdorf K (2012). A lineage of myeloid cells independent of Myb and hematopoietic stem cells. Science.

[106] Dzierzak E, Bigas A (2018). Blood development: hematopoietic stem cell dependence and independence. Cell Stem Cell.

[107] Alsinet C, Primo M, Lorenzi V, Knights AJ, Sancho-Serra C, Park JE (2021). Robust temporal map of human in vitro myelopoiesis using single-cell genomics. BioRxiv Preprint.

[108] Ackermann M, Haake K, Kempf H, Kaschutnig P, Weiss AC, Nguyen A (2021). A 3D iPSC-differentiation model identifies interleukin-3 as a regulator of early human hematopoietic specification. Haematologica.

[109] Galat Y, Dambaeva S, Elcheva I, Khanolkar A, Beaman K, Iannaccone PM (2017). Cytokine-free directed differentiation of human pluripotent stem cells efficiently produces hemogenic endothelium with lymphoid potential. Stem Cell Res Ther.

[110] Sturgeon CM, Ditadi A, Awong G, Kennedy M, Keller G (2014). Wnt signaling controls the specification of definitive and primitive hematopoiesis from human pluripotent stem cells. Nat Biotechnol.

[111] Ginhoux F, Greter M, Leboeuf M, Nandi S, See P, Gokhan S (2010). Fate mapping analysis reveals that adult microglia derive from primitive macrophages. Science.

[112] Hashimoto D, Chow A, Noizat C, Teo P, Beasley MB, Leboeuf M (2013). Tissue-resident macrophages self-maintain locally throughout adult life with minimal contribution from circulating monocytes. Immunity.

[113] Guilliams M, De Kleer I, Henri S, Post S, Vanhoutte L, De Prijck S (2013). Alveolar macrophages develop from fetal monocytes that differentiate into long-lived cells in the first week of life via GM-CSF. J Exp Med.

[114] Litvack ML, Wigle TJ, Lee J, Wang J, Ackerley C, Grunebaum E, Post M (2016). Alveolar-like stem cell-derived myb(-) macrophages promote recovery and survival in airway disease. Am J Respir Crit Care Med.

[115] Lachmann N, Happle C, Ackermann M, Lüttge D, Wetzke M, Merkert S (2014). Gene correction of human induced pluripotent stem cells repairs the cellular phenotype in pulmonary alveolar proteinosis. Am J Respir Crit Care Med.

[116] Kuhn A, Ackermann M, Mussolino C, Cathomen T, Lachmann N, Moritz T (2017). TALEN-mediated functional correction of human iPSC-derived macrophages in context of hereditary pulmonary alveolar proteinosis. Sci Rep.

[117] Mucci A, Lopez-Rodriguez E, Hetzel M, Liu S, Suzuki T, Happle C (2018). iPSC-derived macrophages effectively treat pulmonary alveolar proteinosis in Csf2rb-deficient mice. Stem Cell Rep.

[118] Mantovani A, Marches F, Malesc A, Laghi L, Allavena P (2017). Tumour-associated macrophages as treatment targets in oncology. Nat Rev Clin Oncol.

[119] Zhou J, Tang Z, Gao S, Li C, Feng Y, Zhou X (2020). Tumor-associated macrophages: recent insights and therapies. Front Oncol.

[120] Anderson NR, Minutolo NG, Gill S, Klichinsky M (2021). Macrophage-based approaches for cancer immunotherapy. Cancer Res.

[121] Ritchie D, Mileshkin L, Wall D, Bartholeyns J, Thompson M, Coverdale J (2007). In vivo tracking of macrophage activated killer cells to sites of metastatic ovarian carcinoma. Cancer Immunol Immunother.

[122] Andreesen R, Hennemann B, Krause SW (1998). Adoptive immunotherapy of cancer using monocyte-derived macrophages: rationale, current status, and perspectives. J Leukoc Biol.

[123] Brempelis KJ, Cowan CM, Kreuser SA, Labadie KP, Prieskorn BM, Lieberman NAP (2020). Genetically engineered macrophages persist in solid tumors and locally deliver therapeutic proteins to activate immune responses. J Immunother Cancer.

[124] Moyes KW, Lieberman NA, Kreuser SA, Chinn H, Winter C, Deutsch G (2017). Genetically engineered macrophages: a potential platform for cancer immunotherapy. Hum Gene Ther.

[125] Sloas C, Gill S, Klichinsky M (2021). Engineered CAR-macrophages as adoptive immunotherapies for solid tumors. Front Immunol.

[126] Klichinsky M, Ruella M, Shestova O, Lu XM, Best A, Zeeman M (2020). Human chimeric antigen receptor macrophages for cancer immunotherapy. Nat Biotechnol.

[127] Morrissey MA, Williamson AP, Steinbach AM, Roberts EW, Kern N, Headley MB, Vale RD (2018). Chimeric antigen receptors that trigger phagocytosis. Elife.

[128] Keller AA, Maeß MB, Schnoor M, Scheiding B, Lorkowski S (2018). Transfecting macrophages. Methods Mol Biol.

[129] Burke B, Sumner S, Maitland N, Lewis CE (2002). Macrophages in gene therapy: cellular delivery vehicles and in vivo targets. J Leukoc Biol.

[130] Koba C, Haruta M, Matsunaga Y, Matsumura K, Haga E, Sasaki Y (2013). Therapeutic effect of human iPS-cell-derived myeloid cells expressing IFN-β against peritoneally disseminated cancer in xenograft models. PLoS ONE.

[131] Senju S, Koba C, Haruta M, Matsunaga Y, Matsumura K, Haga E (2014). Application of iPS cell-derived macrophages to cancer therapy. Oncoimmunol.

[132] Miyashita A, Fukushima S, Nakahara S, Kubo Y, Tokuzumi A, Yamashita J (2016). Immunotherapy against metastatic melanoma with human iPS cell-derived myeloid cell lines producing type i interferons. Cancer Immunol Res.

[133] Zhang L, Tian L, Dai X, Yu H, Wang J, Lei A (2020). Pluripotent stem cell-derived CAR-macrophage cells with antigen-dependent anti-cancer cell functions. J Hematol Oncol.

[134] Poltavets AS, Vishnyakova PA, Elchaninov AV, Sukhikh GT, Fatkhudinov TK (2020). Macrophage modification strategies for efficient cell therapy. Cells.

[135] Wang Y, Wang YP, Zheng G, Lee VWS, Ouyang L, Chang DHH (2007). Ex vivo programmed macrophages ameliorate experimental chronic inflammatory renal disease. Kidney Int.

[136] Du Q, Tsuboi N, Shi Y, Ito S, Sugiyama Y, Furuhashi K (2016). Transfusion of CD206D M2 macrophages ameliorates antibody-mediated glomerulonephritis in mice. Am J Pathol.

[137] Mao R, Wang C, Zhang F, Zhao M, Liu S, Liao G (2020). Peritoneal M2 macrophage transplantation as a potential cell therapy for enhancing renal repair in acute kidney injury. J Cell Mol Med.

[138] Knoller N, Auerbach G, Fulga V, Zelig G, Attias J, Bakimer R (2005). Clinical experience using incubated autologous macrophages as a treatment for complete spinal cord injury: phase I study results. J Neurosurg Spine.

[139] Chen J, Wu Y, Duan FX, Wang SN, Guo XY, Ding SQ (2019). Effect of M2 macrophage adoptive transfer on transcriptome profile of injured spinal cords in rats. Exp Biol Med (Maywood).

[140] Shen Y, Song J, Wang Y, Chen Z, Zhang L, Yu J (2019). M2 macrophages promote pulmonary endothelial cells regeneration in sepsis-induced acute lung injury. Ann Transl Med..

[141] Jetten N, Verbruggen S, Gijbels MJ, Post MJ, De Winther MP, Donners MM (2014). Anti-inflammatory M2, but not pro-inflammatory M1 macrophages promote angiogenesis in vivo. Angiogenesis.

[142] Zuloff-Shani A, Adunsky A, Even-Zahav A, Semo H, Orenstein A, Tamir J (2010). Hard to heal pressure ulcers (stage III-IV): efficacy of injected activated macrophage suspension (AMS) as compared with standard of care (SOC) treatment controlled trial. Arch Gerontol Geriatr.

[143] Krzyszczyk P, Schloss R, Palmer A, Berthiaume F (2018). The role of macrophages in acute and chronic wound healing and interventions to promote pro-wound healing phenotypes. Front Physiol.

[144] Ding Y, Zhang D, Wang S, Zhang X, Yang J (2021). Hematogenous macrophages: a new therapeutic target for spinal cord injury. Front Cell Dev Biol.

[145] Alagesan S, Griffin MD (2014). Alternatively activated macrophages as therapeutic agents for kidney disease: in vivo stability is a key factor. Kidney Int.

[146] Cao Q, Wang Y, Zheng D, Sun Y, Wang C, Wang XM (2014). Failed renoprotection by alternatively activated bone marrow macrophages is due to a proliferation-dependent phenotype switch in vivo. Kidney Int.

[147] Fraser AR, Pass C, Burgoyne P, Atkinson A, Bailey L, Laurie A (2017). Development, functional characterization and validation of methodology for GMP-compliant manufacture of phagocytic macrophages: a novel cellular therapeutic for liver cirrhosis. Cytotherapy.

[148] Lopez-Yrigoyen M, Lopez-Yrigoyen M, Fidanza A, Cassetta L, Axton RA, Taylor AH (2018). A human iPSC line capable ofdifferentiating into functional macrophagesexpressing ZsGreen: a tool for the study andin vivotracking of therapeutic cells. Philos Trans R Soc Lond B Biol Sci.

[149] Haideri SS, McKinnon AC, Taylor AH, Kirkwood P, Starkey Lewis PJ, O’Duibhir E (2017). Injection of embryonic stem cell derived macrophages ameliorates fibrosis in a murine model of liver injury. NPJ Regen Med.

[150] Pouyanfard S, Meshgin N, Cruz LS, Diggle K, Hashemi H, Pham TV (2021). Human induced pluripotent stem cell-derived macrophages ameliorate liver fibrosis. Stem Cells.

[151] Jeon OH, Panicker LM, Lu Q, Chae JJ, Feldman RA, Elisseeff JH (2016). Human iPSC-derived osteoblasts and osteoclasts together promote bone regeneration in 3D biomaterials. Sci Rep.

[152] Tasnim F, Xing J, Huang X, Mo S, Wei X, Tan MH (2019). Generation of mature kupffer cells from human induced pluripotent stem cells. Biomaterials.

[153] Mass E, Lachmann N (2021). From macrophage biology to macrophage-based cellular immunotherapies. Gene Ther.

[154] Moroni F, Dwyer BJ, Graham C, Pass C, Bailey L, Ritchie L (2019). Safety profile of autologous macrophage therapy for liver cirrhosis. Clin Ttial.

[155] Yamanaka S (2020). Pluripotent stem cell-based cell therapy-promise and challenges. Cell Stem Cell.

[156] Happle C, Lachmann N, Ackermann M, Mirenska A, Göhring G, Thomay K (2018). Pulmonary transplantation of human induced pluripotent stem cell-derived macrophages ameliorates pulmonary alveolar proteinosis. Am J Respir Crit Care Med.

[157] Bedel A, Beliveau F, Lamrissi-Garcia I, Rousseau B, Moranvillier I, Rucheton B (2017). Preventing pluripotent cell teratoma in regenerative medicine applied to hematology disorders. Stem Cells Transl Med.

[158] Nagashima T, Shimizu K, Matsumoto R, Honda H (2018). Selective elimination of human induced pluripotent stem cells using medium with high concentration of L-alanine. Sci Rep.

[159] Hockemeyer D, Jaenisch R (2016). Induced pluripotent stem cells meet genome editing. Cell Stem Cell.

[160] Lino CA, Harper JC, Carney JP, Timlin JA (2018). Delivering CRISPR: a review of the challenges and approaches. Drug Deliv.

[161] Chakraborty S (2019). Sequencing data from massachusetts general hospital shows Cas9 integration into the genome, highlighting a serious hazard in gene-editing therapeutics [version 1; peer review: 1 approved with reservations]. F1000Res.

[162] Vicente MM, Chaves-Ferreira M, Jorge JMP, Proença JT, Barreto VM (2021). The off-targets of clustered regularly interspaced short palindromic repeats gene editing. Front Cell Dev Biol.

[163] Taylor CJ, Peacock S, Chaudhry AN, Bradley JA, Bolton EM (2012). Generating an iPSC bank for HLA-matched tissue transplantation based on known donor and recipient HLA types. Cell Stem Cell.

[164] Kaneko S, Yamanaka S (2013). To be immunogenic, or not to be: that's the iPSC question. Cell Stem Cell.

[165] Haga E, Endo Y, Haruta M, Koba C, Matsumura K, Takamatsu K (2014). Therapy of peritoneally disseminated colon cancer by TAP-deficient embryonic stem cell-derived macrophages in allogeneic recipients. J Immunol.

[166] Flahou C, Morishima T, Takizawa H, Sugimoto N (2021). Fit-for-all iPSC-derived cell therapies and their evaluation in humanized mice with NK cell immunity. Front Immunol.

[167] Han X, Wang M, Duan S, Franco PJ, Kenty JH, Hedrick P (2019). Generation of hypoimmunogenic human pluripotent stem cells. Proc Natl Acad Sci U S A.

[168] Koga K, Wang B, Kaneko S (2020). Current status and future perspectives of HLA-edited induced pluripotent stem cells. Inflamm Regen.

[169] Guha P, Morgan JW, Mostoslavsky G, Rodrigues NP, Boyd AS (2013). Lack of immune response to differentiated cells derived from syngeneic induced pluripotent stem cells. Cell Stem Cell.

[170] Araki R, Uda M, Hoki Y, Sunayama M, Nakamura M, Ando S (2013). Nature.

[171] Jha BS, Farnoodian M, Bharti K (2021). Regulatory considerations for developing a phase I investigational new drug application for autologous induced pluripotent stem cells-based therapy product. Stem Cells Transl Med.

